# DDX49 is an RNA helicase that affects translation by regulating mRNA export and the levels of pre-ribosomal RNA

**DOI:** 10.1093/nar/gky231

**Published:** 2018-03-30

**Authors:** Sharad Awasthi, Mamta Verma, Arun Mahesh, Mohd Imran K. Khan, Gayathri Govindaraju, Arumugam Rajavelu, Pavithra L Chavali, Sreenivas Chavali, Arunkumar Dhayalan

**Affiliations:** 1 Department of Biotechnology, Pondicherry University, Puducherry 605 014, India; 2 Bacterial and Parasite Disease Biology, Rajiv Gandhi Center for Biotechnology, Trivandrum 695 014, India; 3 MRC Laboratory of Molecular Biology, Francis Crick Avenue, Cambridge Biomedical Campus, Cambridge CB2 0QH, UK

## Abstract

Among the proteins predicted to be a part of the DExD box RNA helicase family, the functions of DDX49 are unknown. Here, we characterize the enzymatic activities and functions of DDX49 by comparing its properties with the well-studied RNA helicase, DDX39B. We find that DDX49 exhibits a robust ATPase and RNA helicase activity, significantly higher than that of DDX39B. DDX49 is required for the efficient export of poly (A)^+^ RNA from nucleus in a splicing-independent manner. Furthermore, DDX49 is a resident protein of nucleolus and regulates the steady state levels of pre-ribosomal RNA by regulating its transcription and stability. These dual functions of regulating mRNA export and pre-ribosomal RNA levels enable DDX49 to modulate global translation. Phenotypically, DDX49 promotes proliferation and colony forming potential of cells. Strikingly, DDX49 is significantly elevated in diverse cancer types suggesting that the increased abundance of DDX49 has a role in oncogenic transformation of cells. Taken together, this study shows the physiological role of DDX49 in regulating distinct steps of mRNA and pre-ribosomal RNA metabolism and hence translation and potential pathological role of its dysregulation, especially in cancers.

## INTRODUCTION

The DExD RNA helicases belong to Super Family 2 of helicases and are involved in nearly all aspects of RNA metabolism (1,2). DExD RNA helicases contain a conserved core, which consists of nine conserved motifs, including the characteristic D-E-x-D motif. These motifs confer ATP hydrolysis and RNA unwinding activities that establish them as RNA helicases (2,3). Many of these helicases are components of multi-protein complexes and they participate in a variety of cellular functions ranging from splicing, nuclear export of mRNAs and mRNA decay to translation and ribosome biogenesis (1–3). For instance, the RNA helicase DDX48 (eIF4AIII), a component of exon junction complex, is essential for nonsense-mediated RNA decay (4–7). Similarly, DDX39B is an essential splicing factor, which promotes the recruitment of U2 snRNP to the pre-mRNA and unwinding of U4/U6 snRNA duplex (8,9). Additionally, DDX39B recruits the mRNA export adaptor proteins, Aly and UIF to the mRNAs and facilitates the export of mRNAs to the cytoplasm (10–13). Besides DDX39B, other RNA helicases such as DDX19 and DDX25 (Dbp5 in *Saccharomyces cerevisiae*) are also involved in nuclear-cytoplasmic mRNA export (1,14,15). Another related RNA helicase, DDX2A (eIF4A) is required for the formation of translation initiation complex (1). Although the members of DExD family of RNA helicases share conserved motifs, specific member(s) regulate distinct steps of RNA metabolism.

Among the 38 members of human DExD RNA helicases (Supplementary Table S1, Supplementary Figure S1), only few of them have been extensively characterized for their molecular functions. DDX49 is one such uncharacterized RNA helicase (16), which has been implicated in viral infections and breast cancers in high throughput screens, suggesting an important physiological role (17–20). However, very little is known about its enzymatic activities, sub-cellular localization, cellular functions and physiological roles. In this study, we extensively characterized the physiological functions of DDX49 by comparing its properties with a well-characterized RNA helicase DDX39B (8–13,21–24). We provide evidence that DDX49 displays a robust ATPase and RNA helicase activity and it is involved in the export of poly (A)^+^ mRNAs to cytoplasm. We find that DDX49 is localized in nucleolus and regulates the steady state levels of pre-ribosomal 47S RNA and global translation. Furthermore, DDX49 affects cell proliferation and its aberrant expression might have oncogenic potential.

## MATERIALS AND METHODS

### Cloning, expression and purification

The sequence encoding full length human DDX49 (NM_019070.3) and DDX39B (NM_004640.6) were PCR amplified from cDNA prepared from HEK293 cells and cloned in pGEX-6P2 vector (GE Healthcare) using the restriction sites EcoRI and XhoI (DDX49) and BamHI and XhoI (DDX39B) to generate pGEX-DDX49 and pGEX-DDX39B constructs respectively. DDX49 and DDX39B were also subcloned in pEGFP-C1 vector (Clontech) using the restriction sites EcoRI and KpnI (DDX49) and BamHI and XhoI (DDX39B) to generate pEGFP-DDX49 and pEGFP-DDX39B constructs respectively.

**Figure 1.**
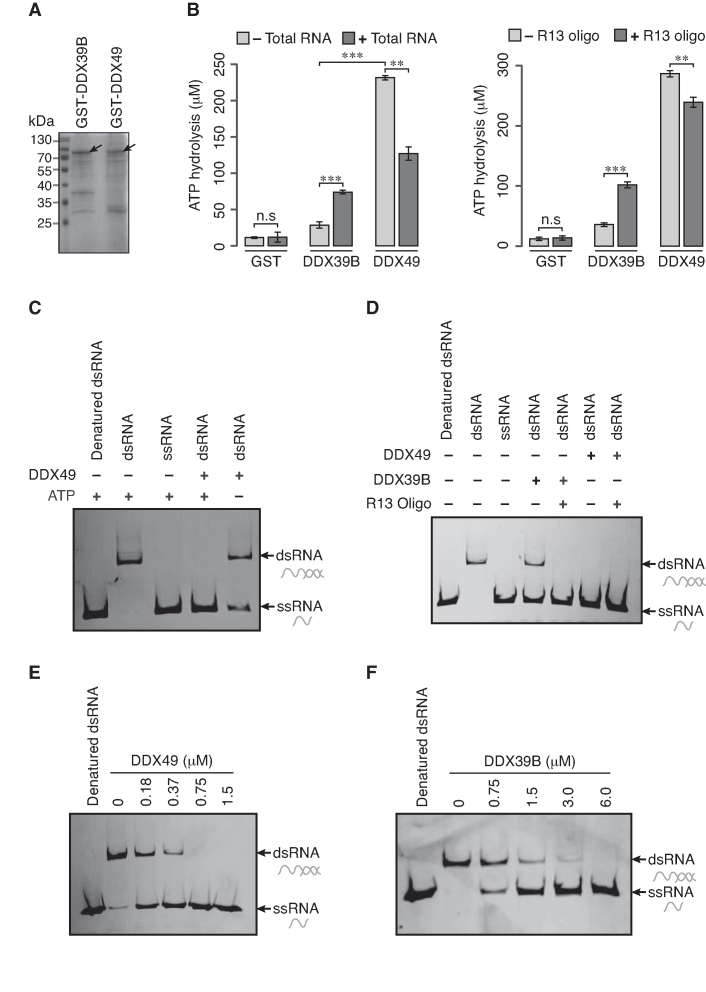
DDX49 is an ATP dependent RNA helicase. (**A**) Coomassie stained gel of purified GST tagged DDX39B and GST tagged DDX49. The arrow head indicates the full-length proteins. The predicted molecular size of GST-tagged DDX39B is 76.1 kDa and the GST-tagged DDX49 is 81.2 kDa. (**B**) The ATPase activity of the purified DDX49 and DDX39B with or without total RNA (left panel) or R13 RNA oligo (right panel). The ATP hydrolysis was assessed with the malachite green-molybdate reagent. Data are represented as mean of three independent experiments, with error bars representing standard deviations. Statistical significance was assessed using a two-tailed Welch's unequal variance t-test. n.s stands for not significant, **P< 0.01 and ***P< 0.001. (**C**) RNA helicase activity of DDX49 in the presence and absence of ATP. (**D**) RNA helicase activity of DDX49 and DDX39B in the presence and absence of R13 RNA oligo. RNA helicase activity of (**E**) DDX49 and (**F**) DDX39B, in the absence of R13 oligo is dependent on the concentration of respective proteins. The helicase assays were repeated at least twice to confirm the reproducibility. dsRNA stands for duplex RNA oligo prepared from labeled and unlabeled RNA oligos; ssRNA for single stranded labeled RNA oligo. dsRNA was incubated at 100°C for 10 min (Denatured dsRNA) prior to loading onto gel.

For overexpression of GST-tagged DDX49 and DDX39B, *E. coli* BL21 carrying the corresponding plasmid construct were grown in the Luria-Bertani medium at 37°C to OD_600_ ≈ 0.6, then shifted to 22°C for 20 min and induced overnight with 1 mM isopropyl-β-d-thiogalactoside. The cells were collected by centrifugation and resuspended in 20 mM HEPES (pH 7.5), 0.5 M KCl, 0.2 mM DTT, 1 mM EDTA and 10% glycerol and disrupted by sonication. The supernatants were passed through glutathione Sepharose 4B resin (GE Healthcare) and washed with same buffer. The bound proteins were eluted with similar buffer containing 40 mM glutathione and dialyzed in 20 mM HEPES (pH 7.5), 0.2 M KCl, 0.2 mM DTT, 1 mM EDTA and 10% glycerol and stored at -80°C. The protein concentrations were quantified by BCA assay and by measuring the absorbance of the purified proteins at 280 nm. The purified proteins were loaded in 12% SDS PAGE to analyze quality and quantity of the proteins (Figure 1A).

### ATPase assay

The ATPase activity of DDX49 or DDX39B was measured by incubating the 1 μM of DDX49 or DDX39B in a 50 μl reaction mixture containing 50 mM HEPES (pH 6.5), 50 mM KCl, 2 mM MgCl_2_, 0.1 mg/ml BSA, 1 mM DTT, 500 μM ATP with or without 15 μg of total RNA from HEK293 cells or R13 oligo (5′GCU UUA CGG UGC U3′) at 37°C for 1 h. The reactions were terminated by adding 5 μl of 0.5 M EDTA and 150 μl of malachite green/molybdate reagent (25). The amount of phosphate released in the ATPase assay was quantified by measuring the O.D. at 655 nm and its concentration was interpolated from a standard curve of inorganic phosphate (KH_2_PO_4_).

### Helicase assay

To study the RNA helicase activity of DDX49 or DDX39B, the HPLC purified single stranded 10-mer RNA oligo (5′-GCU UUA CGG U-3′) labeled with Alexa Flour 488 at 5′ end and the 40-mer RNA unlabeled oligo (5′-AAA AAC AAA ACA AAA CAA AAC AAA ACU AGC ACC GUA AAG C-3′) were procured from Eurogentec. The labeled and unlabeled RNA oligos were annealed in the molar ratio of 1:1.25 in annealing buffer (10 mM Tris–HCl (pH 7.4), 1 mM EDTA and 100 mM NaCl) to prepare a double stranded RNA oligo substrate for helicase assay.

Helicase assay was performed as described previously with minor modifications (26). Briefly, the DDX49 or DDX39B protein was incubated in 20 μl of helicase reaction buffer containing 70 mM Tris (pH 7.5), 200 mM NaCl, 2 mM MgCl_2,_ 2 mM DTT, 2 mM ATP, 10 nM double stranded RNA oligo substrate and 40 units of RNaseOUT inhibitor (ThermoFisher Scientific) with or without 0.5 μM of R13 oligo at 37°C for 30 min. The reactions were terminated by adding the stop solution (100 mM EDTA, 0.5% SDS, 0.1% bromophenol blue, 0.1% xylene cyanol and 50% glycerol) to the reaction mixture. The final reaction mixtures were loaded onto a 12% native polyacrylamide gel electrophoresed at 4°C. Single- and double-stranded RNAs were visualized using Phosphor Imager.

### Cell culture, transfection and subcellular localization experiments

HEK293, HeLa and U251MG cells were grown in DMEM supplemented with 10% FBS and grown at 37°C under 5% CO_2._ Plasmid based transfections were performed by using the standard calcium phosphate precipitation for HEK293, HeLa cells and Lipofectamine 2000 (Invitrogen) for U251MG cells. siRNA transfections were performed using Lipofectamine 2000 as per the manufacturer's instructions. For the subcellular localization experiments, cells of different types were seeded in coverslips and transfected with pEGFP-DDX39B or pEGFP-DDX49. Twenty four hours post transfection cells were fixed in 4% formaldehyde for 10 min at room temperature and blocked in PBS with 5% BSA. For subcellular localization experiments, cells were directly treated with DAPI and mounted in prolong antifade. For colocalization experiments, fibrillarin antibody (1:1000; Abcam, ab5821) was added for an hour at 37°C. Cells were washed in 1× PBS tween, incubated with rabbit alex fluor 555 antibody for 30 min at 37°C. Subsequently, cells were treated with DAPI and mounted in prolong antifade. Confocal images were acquired using a Zeiss LSM 710 under 63X objective.

### Fluorescent *in situ* hybridization (FISH)

To study the role of DDX49 or DDX39B in the nuclear export of mRNAs, we performed FISH experiments as described previously (27). Briefly, HeLa cells were seeded in cover slips and transfected with control siRNA, DDX39B siRNA or DDX49 siRNA. The siRNAs used in these experiments (Supplementary Table S2) were procured from Eurogentec. After 60 hours of transfection, the cells were fixed with 4% formaldehyde in PBS for 10 min and permeablized with PBS containing 0.3% Triton X-100 for 5 min. After the permeabilization, the cells were washed with 2× SSC buffer (300 mM NaCl and 30 mM sodium citrate) and incubated in 2× SSC buffer containing 1 ng/μl of oligo-dT (50-mer), labelled with ATTO 550 dye at the 5′ end for 16–24 h at 42°C in a humid chamber. After incubation, the cells were washed with 2× SSC buffer twice for 5 min each and once with 0.5× SSC buffer followed by one wash with PBS. Subsequently, cells were treated with DAPI and embedded in Mowiol. Images were acquired using a fluorescence microscope (Nikon-Eclipse 80i) with 100× oil immersion objective, with the continuous exposure time of 200 milliseconds across all samples. The efficiency of knockdown of DDX49 and DDX39B were confirmed by quantitative RT-PCR (qRT-PCR) using specific primers (Supplementary Table S3) and western blotting using DDX49 antibody (Abcam, ab188010) and DDX39B antibody (Abcam, ab181061) respectively.

### Quantitative RT-PCR analysis to study mRNA export

To quantify the cytoplasmic and nuclear levels of EGR1, GAPDH and YY1 transcripts, HeLa cells were transfected with control siRNA or DDX49 siRNA or DDX39B siRNA or pEGFP-DDX49 construct or pEGFP-DDX39B construct. After 60 hours of transfection, the cells were harvested and processed for the preparation of cytoplasmic and total RNAs as described previously (11). The total RNA was isolated from the cells by using Trizol reagent (Life Technologies) as per the manufacturer's instructions. The cytoplasmic fractions were isolated by incubating the cells in hypotonic buffer (10 mM Tris (pH 8.0), 1 mM KCl, 1.5 mM MgCl_2_ and 1 mM DTT) on a spin wheel rotor at 4°C for 15 min. Then the nuclei were pelleted by the centrifugation at 700g for 10 min at 4°C and the supernatant containing the cytoplasmic extracts were collected and centrifuged once again at 16 000g for 1 min to remove the cellular debris. The RNA was extracted from the cytoplasmic fractions by using Trizol reagent (Life Technologies). The cytoplasmic extracts were also analyzed by western blotting using beta actin antibody (Sigma, A2228) or histone 3 antibody (Abcam, ab1791) to rule out nuclear contamination in cytoplasmic extract (Supplementary Figure S7). The total or cytoplasmic RNAs was reverse transcribed using random hexamers and Maxima H Minus Reverse Transcriptase (Fermentas). qRT-PCR analysis of EGR1, GAPDH and YY1 transcripts was performed on a Light cycler real time PCR system (Roche) using Fast start essential DNA green master mix (Roche) as per the manufacturer's instructions. The primers used in the qRT-PCR experiment are listed in Supplementary Table S3.

### Quantitative RT-PCR analysis of pre-ribosomal 47S rRNA

HEK293 cells were transfected with control siRNA or DDX49 siRNA or pEGFP-DDX49 construct. After 48 hours of transfection, the cells were harvested and the qRT-PCR analyses of 47S rRNA were performed as described above. The 47S rRNA primers used in the qRT-PCR experiments are listed in Supplementary Table S3.

### Actinomycin D pulse chase assay

To study the influence of DDX49 on the stability of 47S rRNA, we performed Actinomycin D pulse chase assay. Actinomycin D inhibits synthesis of new RNA molecules. For this, HEK293 cells were transfected with control siRNA or DDX49 siRNA or pEGFP-DDX49 construct. After 48 hours of transfection, 5 μg/ml of actinomycin D (Sigma) was added and cells were harvested at different time points (0, 5, 15, 30 and 45 min). Total RNA was isolated and the qRT-PCR analyses of 47S rRNA were performed as described above.

### 5-Fluorouridine pulse chase assay

To study the role of DDX49 on the transcription of pre-ribosomal RNA, HeLa cells were seeded in the coverslips and transfected with control siRNA or DDX49 siRNA. After 48 h of transfection, the cells were pulse labeled with 2 mM of 5-fluorouridine (FUrd) (Sigma) for 20 min. After the incubation, the cells were fixed with 2% formaldehyde for 10 min and permeablized with 0.5% Triton X-100 for 10 min. The cells were then blocked with 1% BSA in PBST (PBS containing 0.1% Tween 20) for one hour and probed with BrdU antibody (Sigma, Cat. No. B8434) overnight at 4°C. After washing with PBS, the cells were incubated with FITC conjugated secondary antibody (Sigma, Cat. No. F2012) for 1 h at room temperature. After washing with PBS, the cells were treated with DAPI and embedded in Mowiol and images were taken in fluorescence microscope (Nikon Eclipse Ti) using 63× oil immersion objective.

### Chromatin immunoprecipitation

To identify the recruitment of DDX49 to rDNA promoter and regulatory regions, we performed chromatin immunoprecipitation experiment. For this, HEK293 cells were treated with formaldehyde to the final concentration of 1% and incubated at room temperature for 10 min to facilitate cross linking. Glycine was added to the final concentration of 125 mM and incubated for 5 min to quench the cross-linking. The cells were then re-suspended in a hypotonic buffer (10 mM Tris (pH 8.0), 1 mM KCl, 1.5 mM MgCl_2,_ 1 mM DTT and Protease Inhibitor Cocktail (Roche)) and incubated on rotator at 4°C for 1 h to promote the hypotonic lysis. The nuclei were then pelleted by centrifugation at 10 000g at 4°C for 10 min and re-suspended in buffer N (15 mM Tris (pH 7.5), 15 mM NaCl, 60 mM KCl, 250 mM sucrose, 5 mM MgCl_2_ and 1 mM CaCl_2_). The nuclei were subjected to micrococcal nuclease (NEB) digestion at 37°C for 20 min and the digestion was terminated by adding EDTA. After the digestion, the nuclei were re-suspended in ChIP buffer I (25 mM Tris (pH 7.5), 5 mM MgCl_2,_ 100 mM KCl, 10% glycerol and 0.1% NP40) and sonicated for 15 min with 10 s pulse on and 50 s pulse off time using QSonica ultrasonicator fitted with microprobe to break open the nuclei and shear the chromatin. The nuclei lysate was centrifuged at 10 000g at 4°C for 10 min. The supernatant containing the chromatin fraction was used for immunoprecipitation. The fragmentation pattern of prepared chromatin was verified by agarose gel electrophoresis prior to the immunoprecipitation experiment (Supplementary Figure S2) and a portion of chromatin was reserved for the preparation of input DNA.

For immunoprecipitation, 15 μg of rabbit IgG (CST, #2729) or DDX49 antibody (Abcam, ab188010) was incubated with 15 μg of chromatin at 4°C for 1 h. After the incubation, 25 μl of protein A dyna beads (Invitrogen) was added and incubated at 4°C for another 10 h. After the incubation, the beads were washed thrice with ChIP wash buffer II (25 mM Tris (pH 7.5), 5 mM MgCl_2,_ 300 mM KCl, 10% glycerol and 0.1% NP40), once each with LiCl buffer (50 mM HEPES (pH 7.5), 500 mM LiCl, 1 mM EDTA, 0.7% sodium deoxycholate and 1% NP40) and ChIP wash buffer I (25 mM Tris (pH 7.5), 5 mM MgCl_2,_ 100 mM KCl, 10% glycerol and 0.1% NP40). The bound chromatin fraction was eluted by incubating the beads in ChIP elution buffer (50 mM Tris (pH 7.5), 1mM EDTA and 1% SDS) at 65°C for 10 min. The crosslinks were reversed by treating the eluted chromatin with RNase A and Proteinase K and the DNA was purified from the samples using a PCR purification kit (Machery Nagel) according to the manufacturer's instructions. The purified DNA was subjected to quantitative real-time PCR (qPCR) analysis. The qRT-PCR analysis was performed on a Light cycler real time PCR system (Roche) using Fast start essential DNA green master mix (Roche) as per the manufacturer's instructions. The primers used in the qPCR experiments are listed in Supplementary Table S3.

### Puromycin incorporation assay

To investigate the role of DDX49 in translation, we performed puromycin incorporation assay. Puromycin is a structural analog of aminoacyl tRNAs, which is incorporated into the nascent polypeptides and inhibits the elongation step of protein synthesis (28). Hence, the quantification of puromycin incorporated polypeptides provides a measure of the global translation efficiency in the cells (29). On the other hand, cycloheximide inhibits the eukaryotic protein synthesis by inhibiting eEF2-mediated translocation step (30) and hence the cells which are treated with cycloheximide prior to the puromycin addition will be devoid of puromycin.

For this assay, HEK293 cells were transfected with control siRNA or DDX49 siRNA. After 48 h of transfection, the cells were treated with puromycin (Sigma) at the concentration of 10 μg/ml for 15 min. After incubation, the cells were washed with PBS and lysed in a lysis buffer (10 mM Tris (pH 7.5), 150 mM NaCl, 0.5 mM EDTA, 0.5% NP-40 and Protease Inhibitor Cocktail (Roche)). The negative control cells were pre-treated with cyclohexamide (100 μg/ml) for 10 min prior to the addition of puromycin. The cell lysates were loaded in 12% SDS PAGE and transferred to PVDF membrane and probed with puromycin antibody (Merck, MABE343) or beta-actin antibody (Sigma, A2228).

### Cell proliferation assay

To study the influence of DDX49 on cell proliferation, we seeded 8 × 10^3^ HeLa cells in 24-well culture plate and transfected with control siRNA or DDX49 siRNA or pEGFP-C1 vector or pEGFP-DDX49 construct. The cells were harvested at different time points (48, 72 and 96 h) post-transfection and stained with equal volume of 0.5% tryphan blue. The viable cells were counted using hemocytometer.

### MTT assay

To study the effect of DDX49 perturbation on cell proliferation, we also performed MTT assay. For this, we seeded 10 × 10^3^ HeLa cells in 96-well culture plate and transfected with control siRNA or DDX49 siRNA or pEGFP-C1 vector or pEGFP-DDX49 construct or treated with 0.5 μg of MG-132 drug (Sigma). MG-132, a potent proteosomal inhibitor and an inducer of apoptosis (31,32) was used as positive control in the assay. After 72 h of transfection or drug treatment, we performed the MTT assay as described previously (33).

### Clonogenic assay

To study the role of DDX49 on colony forming capacity of the cells, we seeded HeLa cells in 24-well plate and transfected with control siRNA or DDX49 siRNA or pEGFP-C1 vector or pEGFP-DDX49 construct. After 24 h of transfection, 1500 cells were transferred to 35 mm dishes and maintained for 10 days with change of media once in every three days. After 10 days of incubation, cells were fixed in 4% formaldehyde and stained with 2% crystal violet for 30 min and then rinsed with deionized water and left to air dry. The images were captured in gel documentation system (Bio-Rad) and the colony counting was done by using ImageJ software.

### Datasets and computational analyses

We collated 38 DExD family members based on those classified in Uniprot database (34) and literature (35). For identifying conserved positions and putative motifs, we generated the multiple sequence alignment of the 38 DExD family members using the ClustalW option of MAFFT from the EMBL-EBI web server (36). From this, we also computed pairwise sequence similarity of DDX49 with other DExD family members. We retrieved human protein-protein interaction data from BioGRID (37). We used Jaccard Similarity Index (JSI) to quantify the extent of overlap in the interacting partners of DDX49 and each of the other DExD box RNA helicases. The principle behind calculating JSI is provided in Supplementary Figure S10B. JSI can take a value from 0 to 1, where 0 indicates that there is no overlap between DDX49 and a given DExD RNA helicase, while 1 indicates that all interacting proteins are common between the two helicases. Therefore, a smaller JSI indicates little overlap of proteins interacting with DDX49 with other DExD box RNA helicases. We retrieved differential gene-expression data for DDX49 obtained by RNA sequencing in different cancer types with statistical significance estimates corrected for multiple testing using Benjamini-Hochberg method of False Discovery Rates (FDR) from BioXpress database (38). Cancer types, in which less than three patients were analyzed for either DDX39 or DDX49, were disregarded.

## RESULTS

### DDX49 is an ATP dependent RNA helicase

We first investigated if the computationally predicted DExD RNA helicase family member DDX49 has ATPase and RNA helicase activity. We included the well-studied DExD RNA helicase DDX39B, as a positive control for measuring these activities. To this end, we cloned the DDX49 and DDX39B into bacterial expression cassette in frame with GST. The recombinant proteins were over-expressed and affinity purified (Figure 1A). The ATPase activity of DDX49 and DDX39B were analyzed by calorimetric assay using malachite green/molybdate reagent. We found that DDX49 exhibits a robust ATPase activity, which is ∼12 fold higher than that of DDX39B (Figure 1B). The ATPase activity of DDX39B is known to be stimulated by RNA or RNA oligos (22,24). Hence, we investigated the effect of RNA oligo or total RNA purified from HEK293 cells on the ATPase activity of DDX49 and DDX39B. Previously, it was shown that a random oligo sequence (R13) could stimulate the ATPase activity of DDX39B (22). We used this R13 RNA oligo to study its effect on the ATPase activity of DDX49 and compare it with DDX39B. Similarly, we also tested for the effect of total RNA on the ATPase activity of DDX39B and DDX49. As reported previously, we found that the ATPase activity of DDX39B was stimulated by the addition of total RNA or RNA oligo (22,24). In contrast, we found that the addition of RNA oligo or total RNA inhibited the ATPase activity of DDX49 by 17% and 45% respectively (Figure 1B). These findings suggest that the ATPase activities of these helicases might have distinct modes of regulation.

We next investigated the RNA helicase activity of DDX49 by employing a fluorescently labeled double stranded RNA (dsRNA) as substrate. We found that DDX49 unwinds the dsRNA effectively into single stranded RNAs (ssRNAs) and this activity was ATP dependent (Figure 1C). We then examined the helicase activity of DDX49 and DDX39B on dsRNA in the presence of single stranded RNA oligo (ssRNA oligo). We found that the helicase activity of DDX39B was stimulated by the addition of RNA oligo (Figure 1D). However, we could not determine the effect of ssRNA oligo on the helicase activity of DDX49 under these same conditions, as DDX49 unwound the dsRNA substrate completely (Figure 1D). To overcome this, we investigated the effect of RNA oligo or total RNA on the helicase activity of DDX49 by using low concentrations of DDX49 and found that the helicase activity of DDX49 was unaffected by the addition of RNA oligo or total RNA (Supplementary Figure S3). Strikingly, in these assays, we found that 1.5 μM of DDX49 was sufficient to completely unwind the dsRNA. Contrarily, twice the amount of DDX39B (3 μM) could only partially unwind the dsRNA in a similar experimental setup (Figure 1D). To quantify the magnitude of differences in the helicase activity, we performed a protein titration experiment of DDX49 and DDX39B on dsRNA templates. We observed that a minimum of 0.75 μM of DDX49 or 6 μM of DDX39B is required to completely unwind 10 nM of dsRNA. This demonstrates that the helicase activity of DDX49 is ∼ 8 fold higher than that of DDX39B *in vitro* (Figure 1E and F). Interestingly, the endogenous levels of DDX39B transcripts levels are ∼140-fold higher than that of DDX49 in HEK293 cells (Supplementary Figure S4). The higher ATPase and RNA helicase activity of DDX49 might explain the relative low abundance of DDX49 in the cells. Taken together, these results demonstrate that DDX49 is a robust ATPase and RNA helicase.

### DDX49 is involved in mRNA export in a splicing-independent manner

**Figure 2.**
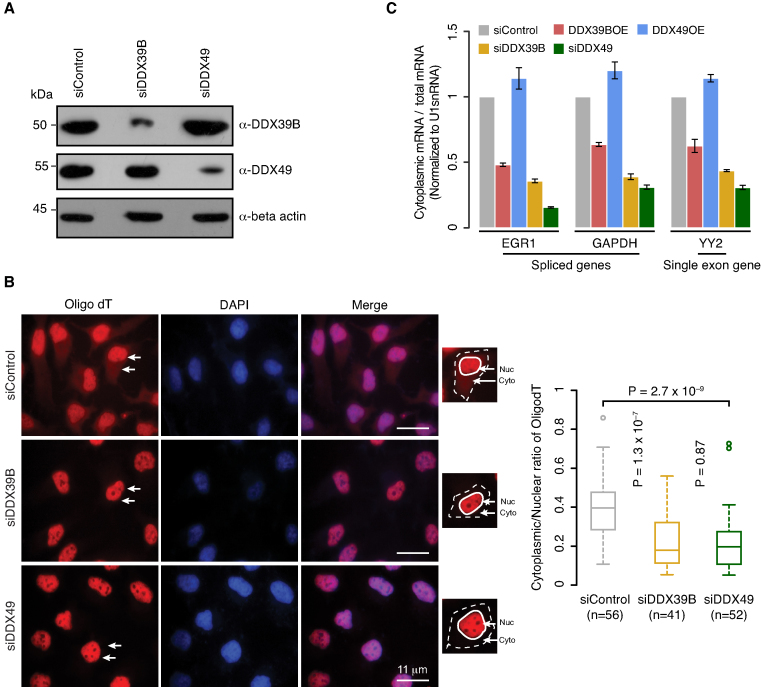
DDX49 is essential for efficient mRNA export. (**A**) Efficiency of siRNA knockdown of DDX39B or DDX49. Western blot analysis of whole cell extracts prepared from the HeLa cells transfected with control siRNA or DDX39B or DDX49 siRNA and probed using indicated antibodies. Beta actin was used as loading control. (**B**) The subcellular distribution of poly (A)^+^ RNA was analyzed by fluorescence in situ hybridization (FISH) using ATTO550 labeled oligo-dT probe in control (scramble siRNA) or DDX39B knockdown or DDX49 knockdown cells. Representative field images are shown in the left panel. Oligo-dT and DAPI staining are shown in red and blue respectively. White arrows depict nucleus and cytoplasm. For each treatment panel, an inset image is provided showing the segmentation of nucleus (continuous white boundary) and cytoplasm (dotted white boundary) used to quantify oligo-dT intensities. Scale bar is depicted. The graph in the right panel represents the quantification of oligo-dT fluorescence signals in at least 40 cells of each group. Fluorescence signals in the nucleus and cytoplasm were quantified using ImageJ software. Statistical significance was assessed using non-parametric Wilcoxon rank-sum test. *P*-Values are depicted. (**C**) Quantitative RT-PCR analysis of the subcellular distribution of mRNAs of the indicated genes in control siRNA, DDX39B overexpression, DDX49 overexpression, DDX39B knockdown and DDX49 knockdown cells. The ratios of cytoplasmic to total mRNA were normalized to U1 small nuclear RNA (snRNA) levels and are presented relative to the control sample. Data are represented as mean of three independent experiments, with error bars representing standard deviations.

A number of the DDX family members such as DDX39B, DDX39A, DDX19, DDX25 and DDX3X are known to be involved in mRNA export (1,12–15,39–41). This prompted us to investigate if DDX49 had a similar function under physiological conditions. To address this, we first knocked down the transcripts of DDX49 and DDX39B in HeLa cells using respective siRNAs. We confirmed the knockdown efficiency of DDX49 and DDX39B both at mRNA and protein levels using quantitative real time PCR (qRT-PCR) and western blotting, respectively (Figure 2A, Supplementary Figure S5). In these knockdown cells, we studied the distribution of poly(A)^+^ RNAs by fluorescence *in situ* hybridization using oligo-dT labeled with ATTO550 fluorescent dye (oligo-dT FISH). Upon the depletion of DDX49 or DDX39B, we observed a significant decrease in cytoplasmic fluorescence, compared to the control siRNA treatment (Figure 2B, Supplementary Figure S6), implying nuclear retention of poly(A)^+^ RNAs. This indicates a prominent role of DDX49, similar to that of DDX39B, in the efficient nuclear export of poly(A)^+^ containing RNAs.

Since, the major population of poly(A)^+^ RNAs represent mRNAs, we next assessed the influence of DDX49 on the nuclear export of mRNAs. To address this, we performed the qRT-PCR analysis of total and cytoplasmic mRNA levels for both spliced and single exon genes upon the knockdown of DDX49 or DDX39B. We ensured that there was no nuclear leakage in the cytoplasmic fractions prior to the isolation of cytoplasmic RNA (Supplementary Figure S7). We observed a very strong reduction of cytoplasmic mRNAs for all the three tested genes upon the depletion of DDX49 corroborating with oligo-dT FISH results (Figure 2C). The reduction in the cytoplasmic mRNA level of the single exon gene, YY2 is similar to the reduction level of spliced genes in DDX49 depleted cells suggesting that the mRNA export function of DDX49, is splicing independent (Figure 2C). In line with previous studies, the knockdown of DDX39B reduced the cytoplasmic levels of mRNAs encoded by both spliced and single exon genes (Figure 2C) (10–13,42). The mRNA export activity of DDX39B has been shown to be a function of its abundance, with increased expression of DDX39B displaying a dominant negative inhibiting effect on mRNA export (12). This prompted us to test if DDX49-mediated mRNA export was also regulated in a concentration-dependent manner. We found that the overexpression of DDX39B blocks the mRNA export (Figure 2C), as reported previously (12). In contrast, the overexpression of DDX49 did not inhibit the export of mRNA. This suggests that DDX49 and DDX39B might regulate distinct steps of mRNA export and/or mediate their effect through different binding partners (Figure 2C). Collectively, these results establish that DDX49 is involved in the export of mRNAs in a splicing independent manner.

### DDX49 is localized in the nucleolus and regulates steady-state levels of pre-ribosomal RNA

**Figure 3.**
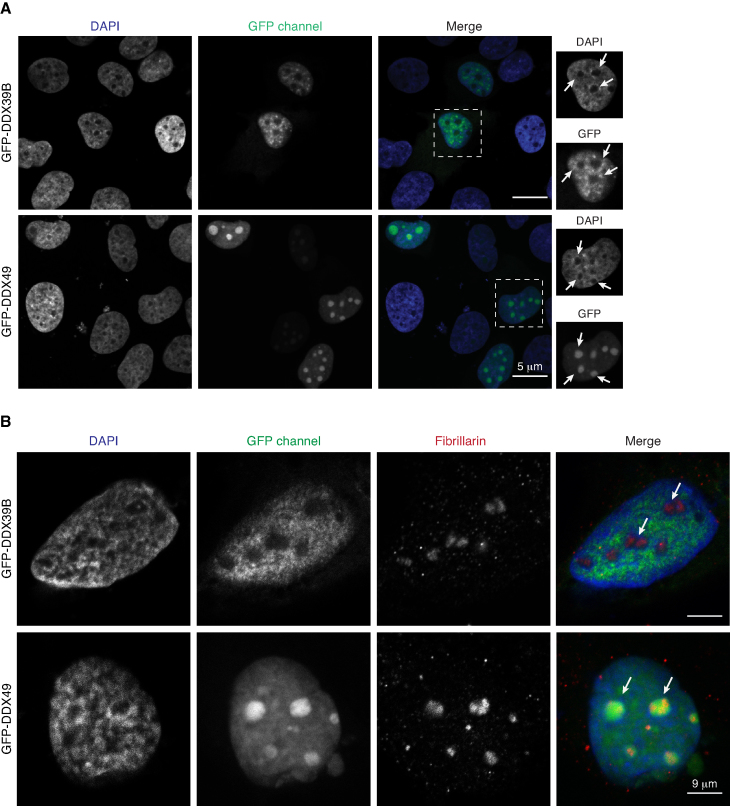
DDX49 is localized in the nucleolus. (**A**) Representative images depicting the subcellular localization of DDX39B or DDX49 in HEK293 cells. Scale bar is depicted. DAPI is shown in blue. Insets show the magnified images of the cells outlined by the dotted white square. The white arrows in the insets indicate the DAPI excluded regions in the nucleus. (**B**) Representative images depicting the colocalization of DDX49 and the nucleolar marker protein fibrillarin. HEK293 cells were transfected with pEGFP-DDX49 or pEGFP-DDX39B and immunostained with fibrillarin antibody (red) which stains the nucleolus. The images show the nucleolar staining of DDX49 and exclusion of DDX39B from nucleolus. The white arrows are used to indicate nucleolus. Scale bar is depicted.

**Figure 4.**
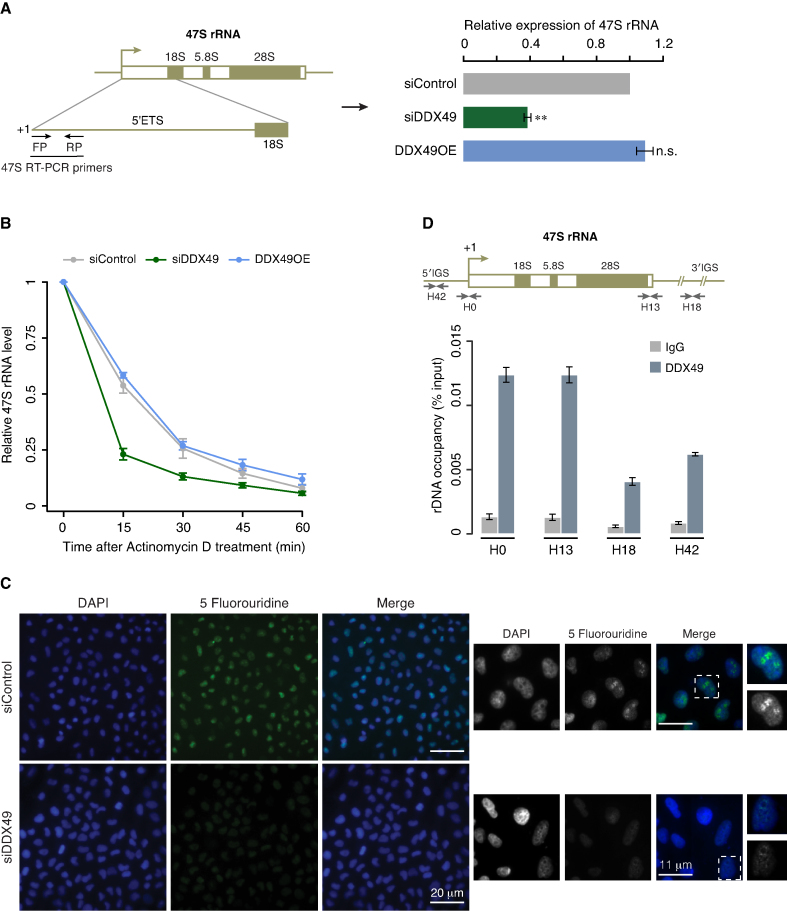
DDX49 is essential for maintaining steady state levels of pre-ribosomal RNA. (**A**) DDX49 is essential for maintaining steady state levels of pre-ribosomal RNA. The schema on the left depicts 47S rRNA gene architecture and the positions of the primers that were used in the quantification of 47S rRNA levels. Quantitative RT-PCR analysis of the pre-ribosomal 47S rRNA levels in control (scrambled siRNA), DDX49 overexpression and DDX49 knockdown cells (right panel). The 47S rRNA levels were normalized to GAPDH expression and are presented relative to the control sample. Data are represented as mean of three independent experiments, with error bars representing standard deviations. Statistical significance of differences in distribution between siControl and siDDX49 or DDX49OE was assessed by two tailed *t*-test. ** indicates *P* < 0.01; n.s. stands for not significant. (**B**) Effect of the DDX49 perturbation on the stability of pre-ribosomal rRNA. Quantitative RT-PCR analysis of the pre-ribosomal 47S rRNA levels in control siRNA or DDX49 overexpression or DDX49 knockdown cells, which were treated with actinomycin D and harvested at the indicated time points after the addition of actinomycin D. The 47S rRNA levels were normalized to GAPDH expression and are presented relative to the 0 min time point sample. Data are represented as mean of three independent experiments, with error bars representing standard deviations. (**C**) Knock down of DDX49 inhibits the pre-ribosomal rRNA synthesis. Left panel depicts a zoomed-out field view of the cells stained with DAPI (blue) and 5-FUrd labeled nascent RNAs (green). The smaller middle panel depicts 2X magnified images of the field. White dotted squares depict the insets provided in the panel on extreme right, where the magnification of a single nucleus is shown. Scale bar is depicted. (**D**) DDX49 occupies the promoter and regulatory regions of rDNA. Cross-linked chromatin were fragmented and prepared from HEK293 cells and immunoprecipitated with rabbit IgG antibody or DDX49 antibody. The precipitated DNA was analyzed for the enrichment of promoter and different regulatory regions by quantitative RT-PCR. The position of the primers and the schema of the rDNA locus is shown above the graph (H42: –924 to –1042 bp; H0: –51 to +32 bp; H13: +12855 to +12970 bp; H18: +18155 to +18280 bp). Data are shown relative to input and the values represent the mean of three independent experiments, with error bars representing standard deviations.

To gain more insights into the functions of DDX49, we studied the subcellular localization of GFP-tagged DDX49 and DDX39B in HEK293 cells. We found that both the proteins were localized predominantly in the nucleus, albeit with distinct staining patterns (Figure 3A). While the DDX49 protein was also found in DAPI-excluded regions, suggesting the possibility of the nucleolar localization, we observed a very weak staining of DDX39B in the DAPI-excluded regions (Figure 3A). We observed a strong co-localization of DDX49 with the nucleolar marker protein, fibrillarin confirming the nucleolar localization of DDX49 in addition to its nucleoplasmic distribution (Figure 3B). To test if the nucleolar localization of DDX49 was cell-type specific, we expressed GFP tagged DDX39B and DDX49 in other cell lines such as cervical cancer derived HeLa and glioblastoma derived U251MG. We confirmed that DDX49 was nucleolar as well as nucleoplasmic in these cell types suggesting that the nucleolar localization of DDX49 is not cell-type specific (Supplementary Figure S8A and B). Since DDX49 displayed nucleolar localization, we hypothesized that it might have a role in regulating pre-ribosomal RNA levels. A high throughput analysis of the DDX family members using lentiviral based RNAi screens, suggested DDX49 as a potential candidate involved in rRNA regulation (43). However, a mechanistic understanding of the role of DDX49 in the regulation of the levels of ribosomal RNA is lacking. To investigate this, we perturbed the levels of DDX49 through siRNA-mediated knockdown or by overexpression. We then quantified pre-ribosomal 47S rRNA in these cells by using quantitative RT-PCR. We observed that the siRNA-mediated depletion of DDX49 reduced the levels of 47S rRNA by ∼60%. However, the overexpression of DDX49 did not exhibit a significant effect (Figure 4A). Taken together, these findings suggest that a substantial fraction of DDX49 is nucleolar and is involved in the regulation of steady-state levels of pre-ribosomal RNA.

### DDX49 regulates the stability and synthesis of pre-ribosomal RNA

To obtain mechanistic insights into how DDX49 regulates pre-ribosomal RNA levels, we investigated its effect on the (i) stability of pre-ribosomal 47S RNA and (ii) synthesis of 47S rRNA. We assessed the stability of 47S rRNA by actinomycin D pulse chase assay and found that the siRNA mediated depletion of DDX49 substantially reduced the stability of 47S rRNA, while the overexpression of DDX49 did not show any alterations (Figure 4B). This suggests that DDX49 is required to stabilize the 47S rRNA. We then investigated the role of DDX49 on the synthesis of 47S rRNA by 5-Fluorouridine (FUrd) incorporation assay. For this, we depleted DDX49 in HeLa cells, followed by treatment with FUrd. Subsequently, we examined the levels of nascent RNA labeled with FUrd by indirect immunofluorescence. In DDX49 depleted cells, we observed a global reduction of the FUrd staining (Figure 4C). However, in control cells prominent FUrd staining was observed in the DAPI-excluded nucleolar regions. Since the pre-ribosomal RNA synthesis is the major constituent of nucleolar transcription (44,45), a reduction of FUrd staining in the nucleoli of DDX49 depleted cells suggests that it is essential for the efficient transcription of pre-ribosomal RNA. Given that DDX49 might be involved in transcription of 47S rRNA, does it bind to the regulatory regions of rDNA locus? To address this we examined the association of DDX49 with the promoter and other regulatory regions of rDNA (46,47) by chromatin immunoprecipitation (ChIP). We found an enrichment of DDX49 in the promoter and regulatory regions of rDNA locus, with more pronounced occupancy at the promoter region (H0) and at the end of 3′-external transcribed spacer (H13) (Figure 4D). This suggests that DDX49 regulates the synthesis of 47S rRNA by binding to the regulatory regions of rDNA locus. Collectively, these data suggest that DDX49 regulates the stability and transcription of pre-ribosomal 47S rRNA.

### DDX49 regulates the global translation

**Figure 5.**
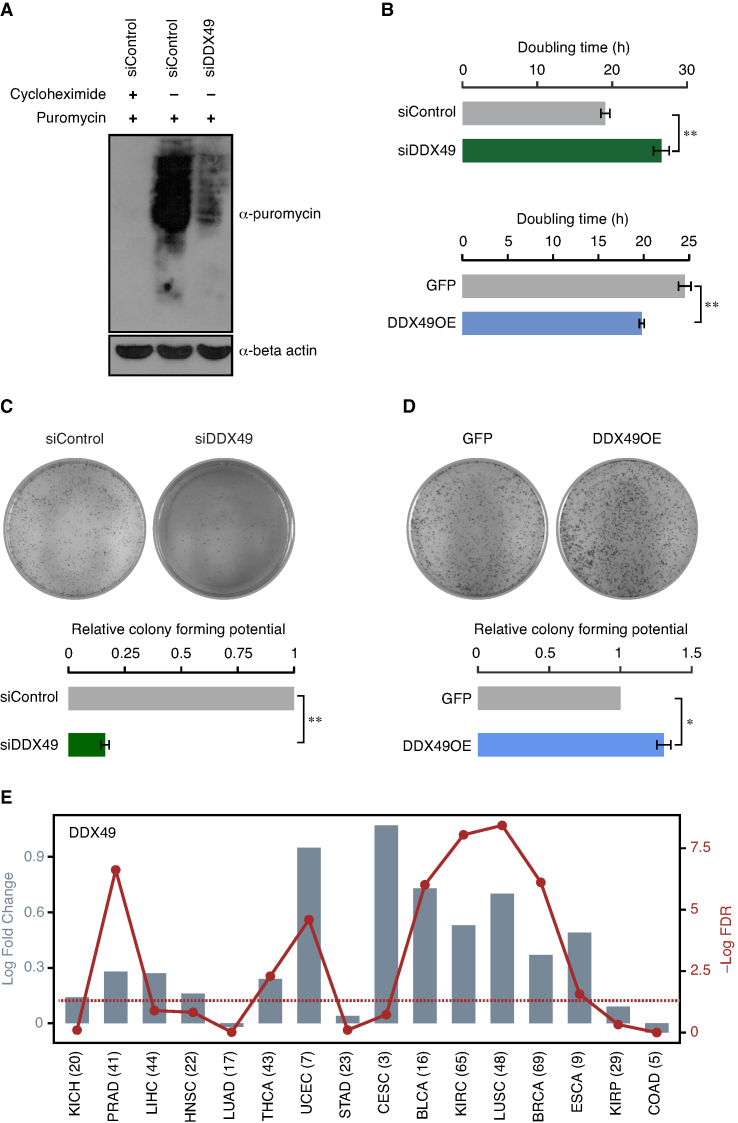
DDX49 regulates the global translation, cell proliferation and clonogenic potential. (**A**) Effect of the DDX49 perturbation on the translation efficiency in the cells. Western blot shows the levels of nascent polypeptides in control (scramble siRNA) or DDX49 knockdown cells, upon puromycin treatment. The first lane represents the negative control, in which the cells were pre-treated with cycloheximide prior to the addition of puromycin. Immunoblotting was performed with puromycin antibody or beta actin antibody. These experiments were done in replicates and representative image is provided here. (**B**) Perturbation of DDX49 affects the cell proliferation. HeLa cells were transfected with control siRNA, DDX49 siRNA, pEGFP-C1 vector or pEGFP-DDX49 construct as indicated and the cell numbers were counted at 48, 72 and 96 hours post transfection (Supplementary Figure S9A and S9B). Doubling time was calculated from cell counts at different time points. The values represent the mean of triplicates, with error bars representing standard deviations. Statistical significance was assessed using two-tailed T-test. ** indicates *P* < 0.01. Perturbation of DDX49 affects the colony forming potential of the cells (panels **C** and **D**). The colony forming potential was analyzed in cells treated with control siRNA, DDX49 siRNA, pEGFP-C1 vector or pEGFP-DDX49 construct as indicated. Colony growth was measured by staining with crystal violet 10 days after transfection. Experiments were performed in triplicates and representative images are provided here. Colony counting was done using ImageJ software and the values in the graph represent the mean of triplicates, with error bars representing standard deviation. Statistical significance was assessed using two-tailed *t*-test. * indicates *P* < 0.05 and ** indicates *P* < 0.01. (**E**) DDX49 is upregulated in diverse cancers. Differential expression of DDX49 in 16 different cancer types is shown as log_2_ fold changes (represented by bars), with each cancer type abbreviated in the X-axis. The red line shows the distribution of –Log_10_ FDR values (False Discovery Rates; *P*-values corrected for multiple testing for each cancer type by Benjamini-Hochberg method), with the dots representing the value for the corresponding cancer type. The dotted line shows the FDR cut-off that corresponds to 0.05, indicating statistical significance. The numbers in the parenthesis represent the number of patients studied for each cancer type. KICH; kidney chromophobe, PRAD; prostate adenocarcinoma, LIHC; liver hepatocellular carcinoma, HNSC; head and neck squamous cell carcinoma, LUAD; lung adenocarcinoma, THCA; thyroid carcinoma, UCEC; uterine corpus endometrial carcinoma, STAD; stomach adenocarcinoma, CESC; cervical squamous cell carcinoma and endocervical adenocarcinoma, BLCA; bladder urothelial carcinoma, KIRC; kidney renal clear cell carcinoma, LUSC; lung squamous cell carcinoma, BRCA; breast invasive carcinoma, ESCA; esophageal carcinoma, KIRP; kidney renal papillary cell carcinoma, COAD; colon adenocarcinoma.

Since DDX49 regulates mRNA export and pre-ribosomal RNA levels, we hypothesized that DDX49 could affect the protein synthesis. To investigate this, we examined the synthesis of nascent polypeptides using puromycin incorporation assay in HEK293 cells in which DDX49 levels were reduced. Puromycin incorporation in polypeptides provides a measure of the global translation efficiency in cells (29). The absence of puromycin signal, in the cells treated with cycloheximide prior to the addition of puromycin, confirmed the reliability of the assay (Figure 5A). The depletion of DDX49 resulted in a strong reduction of puromycin levels compared to the scrambled siRNA treated cells, highlighting the importance of DDX49 in protein synthesis. The role of DDX49 in regulating global translation, might be a cumulative effect of its regulation on mRNA export and pre-ribosomal 47S rRNA levels.

### DDX49 regulates cell proliferation and its elevated levels might promote oncogenesis

Since DDX49 regulates fundamental molecular processes such as regulation of pre-ribosomal RNA levels, mRNA export and protein biosynthesis, we next examined its phenotypic impact by studying its effects on cell proliferation. For this, we perturbed the levels of DDX49 through siRNA-mediated knockdown or overexpression in HeLa cells and analyzed the cell proliferation. We found that the depletion of DDX49 decreased the cell proliferation, as reflected by an increase in doubling time (Figure 5B, Supplementary Figure S9A). In contrast, overexpression of DDX49 increased the cell proliferation (Figure 5B, Supplementary Figure S9B). Interestingly, DDX49 overexpression showed only a subtle increase in mRNA export and pre-ribosomal 47S RNA levels, (Figures 2C and 4A). This suggests that a significant increase in the cell proliferation upon over-expression of DDX49, could either be a cumulative outcome of these subtle effects on RNA metabolism studied here or a result of unidentified functions of DDX49 or both. We confirmed these results using MTT assay, as well (Supplementary Figure S9C). We next assessed the colony forming potential upon knock down or overexpression of DDX49 in HeLa cells using clonogenic assay, which measures the capacity of the single cell to grow into a colony of minimum 50 cells. We observed a very strong reduction in colony forming ability of DDX49 depleted cells suggesting that DDX49 is essential for proper cell growth and proliferation (Figure 5C). Importantly, the overexpression of DDX49 increased the colony forming ability of HeLa cells (Figure 5D). This prompted us to speculate that an increase in the levels of DDX49 could contribute to cancer manifestation. Indeed, we find a significant upregulation of DDX49 in diverse cancer types when we investigated pan cancer differential gene-expression data (Figure 5E). Collectively, these results establish that the DDX49 is essential for the proper cell growth and proliferation and its aberrant elevated expression could contribute to oncogenicity.

## DISCUSSION

In the present study, we show that DDX49 exhibits a robust ATPase and RNA helicase activity and these activities are much higher than DDX39B. The strong catalytic activity of DDX49 might be the reason for the lower cellular abundance of DDX49 compared to the DDX39B (Supplementary Figure S4). Contrary to the RNA-dependent ATPase activity of DDX39B (Figure 1B, ref. (22)), the ATPase activity of DDX49 is inhibited by the addition of RNA oligo or total RNA and the helicase activity of DDX49 is unaffected by the addition of RNA oligo. These data suggest the possibility of (i) RNA stimulation independent activity of DDX49 in the cell and (ii) a possible RNA-based mechanism to negatively regulate the activity of DDX49. This could also imply that DDX39B and DDX49 are recruited in different biochemical contexts.

**Figure 6.**
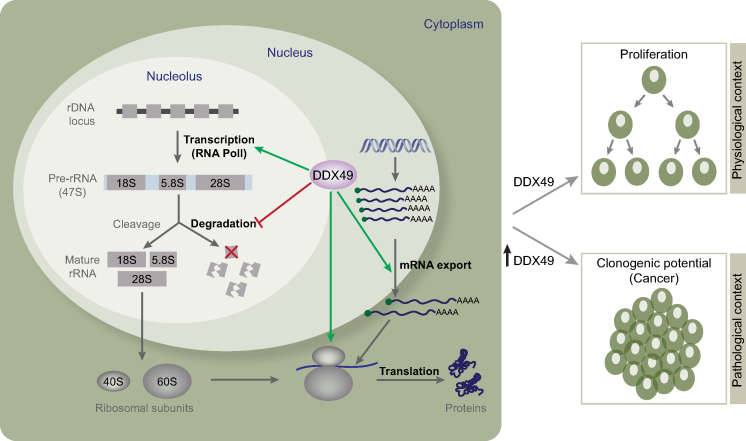
A schematic model illustrating the functional roles of DDX49. DDX49 is involved in the maintenance of the pre-ribosomal 47S RNA levels by enhancing its transcription and stability and is required for the efficient export of mRNAs and global translational regulation. Physiologically DDX49 influences cell growth and proliferation. However, in pathological conditions, especially cancers, elevated levels of DDX49 promote the oncogenic transformation of the cells.

Our *in vivo* studies show that DDX49 is localized in the nucleoplasm and nucleolus and is involved in the regulation of (i) mRNA export and (ii) the transcription and stability of pre-ribosomal, with implications in ribosome biogenesis (Figure 6). Since all these processes are constituent steps in translation, DDX49 also regulates global translation and in turn affects cell growth and proliferation under physiological conditions. Though DDX49 shares some key functionalities such as mRNA export and translational regulation with other DExD RNA helicases, it is very distinct from other helicases. For instance, DDX49 has a modest sequence identity ranging from 24% to 35% with all other DExD RNA helicases, with the exception of DDX47, with which it has 46% sequence identity (Supplementary Figure S10A). Similarly, there is little overlap amongst its protein interaction partners with those of other DExD RNA helicases, as evidenced by the low Jaccard Similarity Index scores (≤0.1; Supplementary Figure S10B). Specifically, DDX49 shares only two of its interaction partners with only 11 out of the 37 other DExD RNA helicases. This implies that 75% of DDX49 interactors (6 out of 8) are unique. The limited number of DDX49 interacting proteins identified so far warrants extensive characterization of its interacting partners and the functional outcome(s) of those interactions. In addition to the functions identified here, DDX49 might also have other regulatory roles such as regulating protein degradation as it interacts with E3 ubiquitin ligase RNF19B and proteasome subunit PSMB2 (Supplementary Figure S10B).

From a pathological standpoint, high throughput screening of interaction partners for HIV proteins identified that the HIV protein, polyprotein Gag interacts with DDX49 (19). Different members of the DExD family have been implicated in viral pathogenesis. For instance, DDX3 and DDX1 are involved in the replication of HIV (48), DDX3, DDX5 and DDX6 play important role in the replication of HCV (49). While the DDX19 facilitates the nuclear export of Influenza A viral mRNAs (50), DDX39B interacts with nucleoprotein of Influenza virus and promotes the viral RNA synthesis (51) and its RNA helicase activity prevents the accumulation of double stranded RNA of Influenza virus, which helps in the evasion of type I interferon response (52). Interestingly, recent studies demonstrated the nucleolar localization of Gag protein and that could be important for nuclear entry of the HIV-1 pre-integration complex (53,54). In the context of the findings presented here, it would be important to delineate the functional role of Gag interaction with the nucleolar protein DDX49 for the better understanding of HIV-1 replication.

Since DDX49 regulates the translational efficiency, this attribute could be potentially hijacked in cancers for promoting uncontrolled cell proliferation. Indeed, the several members of DExD family members show aberrant expression in diverse cancers (55). Elevated expression of DDX49 is observed in breast cancer patients (18). Further, DDX49 seems to promote the stemness of breast cancer stem cells by regulating the expression of proteins such as Oct3/4 and Sox-2 and promoting disease progression (18). In this regard, our findings that DDX49 is upregulated in diverse cancers as well as over-expression of DDX49 promotes proliferation, suggests that DDX49 could have an oncogenic role (Figure 6). Moreover, DDX49 is often mutated in various cancer types (55). We speculate that these mutations might enhance the stability of DDX49 or lead to its gain of function(s), which in turn might contribute to the oncogenicity. Therefore, DDX49 might serve not only as an attractive oncotarget for cancer therapy but also as a potential biomarker for diagnosis and prognosis of various cancers. The interaction of DDX49 with proteins known to be involved in neurological disorders such as Huntingtin-associated protein 1 (HAP1) and Amyloid-beta protein APP suggests that DDX49 might also have a role in diverse proteinopathies (Supplementary Figure S10B). Furthermore, in light of the increasing evidence that dysregulation of mRNA export and translation might play an important role in disease pathogenesis (56,57), extensive studies are required to unravel the mechanisms underlying how dysregulation of DDX49 leads to diseases.

## Supplementary Material

Supplementary Data
