# Supplementary material for: DDX49 is an RNA helicase that affects translation by regulating mRNA export and the levels of pre-ribosomal RNA
Source: Nucleic Acids Res. 2018 Mar 30;46(12):6304–17. doi: 10.1093/nar/gky231 (PMC6158705; doi:10.1093/nar/gky231)
Supplement: Supplementary Data [file gky231_supplemental_files.pdf]

Supplementary information for

DDX49 is an RNA helicase that affects translation by regulating mRNA export and the levels of pre-ribosomal RNA

Sharad Awasthi[1], Mamta Verma[1], Arun Mahesh[1], Mohd. Imran K. Khan[1], Gayathri Govindaraju[2], Arumugam Rajavelu[2], Pavithra L. Chavali[3], Sreenivas Chavali[3*] & Arunkumar Dhayalan[1*]

[1]Department of Biotechnology, Pondicherry University, Puducherry - 605 014, India

[2]Bacterial and Parasite Disease Biology, Rajiv Gandhi Center for Biotechnology, Trivandrum - 695 014, India

[3]MRC Laboratory of Molecular Biology, Francis Crick Avenue, Cambridge Biomedical Campus, Cambridge CB2 0QH, UK

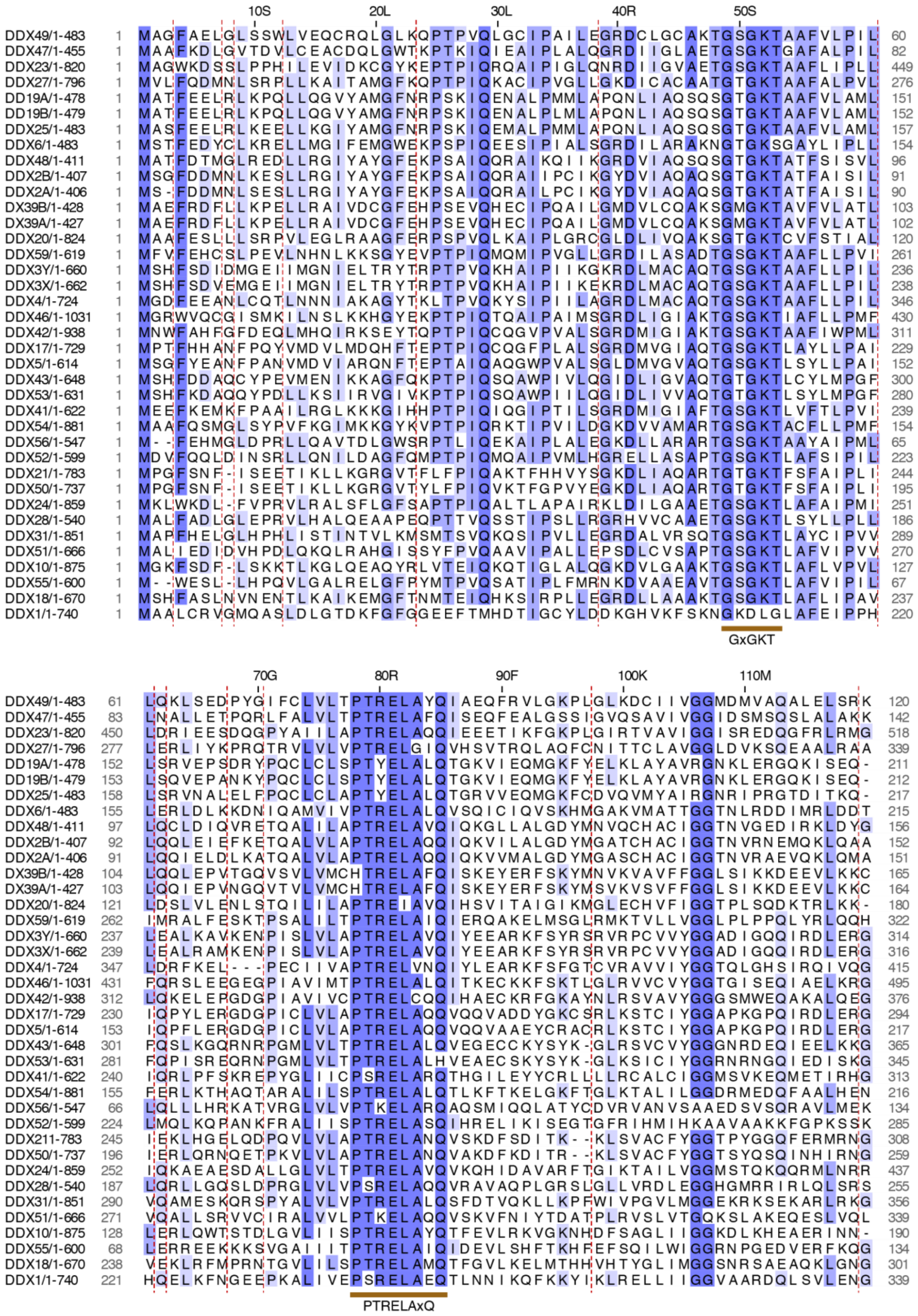
10S
20L
30L
40R
50S
DDX49/1-483
DDX47/1-455
DDX23/1-820
DDX27/1-796
DD19A/1-478
DD19B/1-479
DDX25/1-483
DDX6/1-483
DDX48/1-411
DDX2B/1-407
DDX2A/1-406
DX39B/1-428
DX39A/1-427
DDX20/1-824
DDX59/1-619
DDX3Y/1-660
DDX3X/1-662
DDX4/1-724
DDX46/1-1031
DDX42/1-938
DDX17/1-729
DDX5/1-614
DDX43/1-648
DDX53/1-631
DDX41/1-622
DDX54/1-881
DDX56/1-547
DDX52/1-599
DDX21/1-783
DDX50/1-737
DDX24/1-859
DDX28/1-540
DDX31/1-851
DDX51/1-666
DDX10/1-875
DDX55/1-600
DDX18/1-670
DDX1/1-740
GxGKT
70G
80R
90F
100K
110M
PTRELAxQ

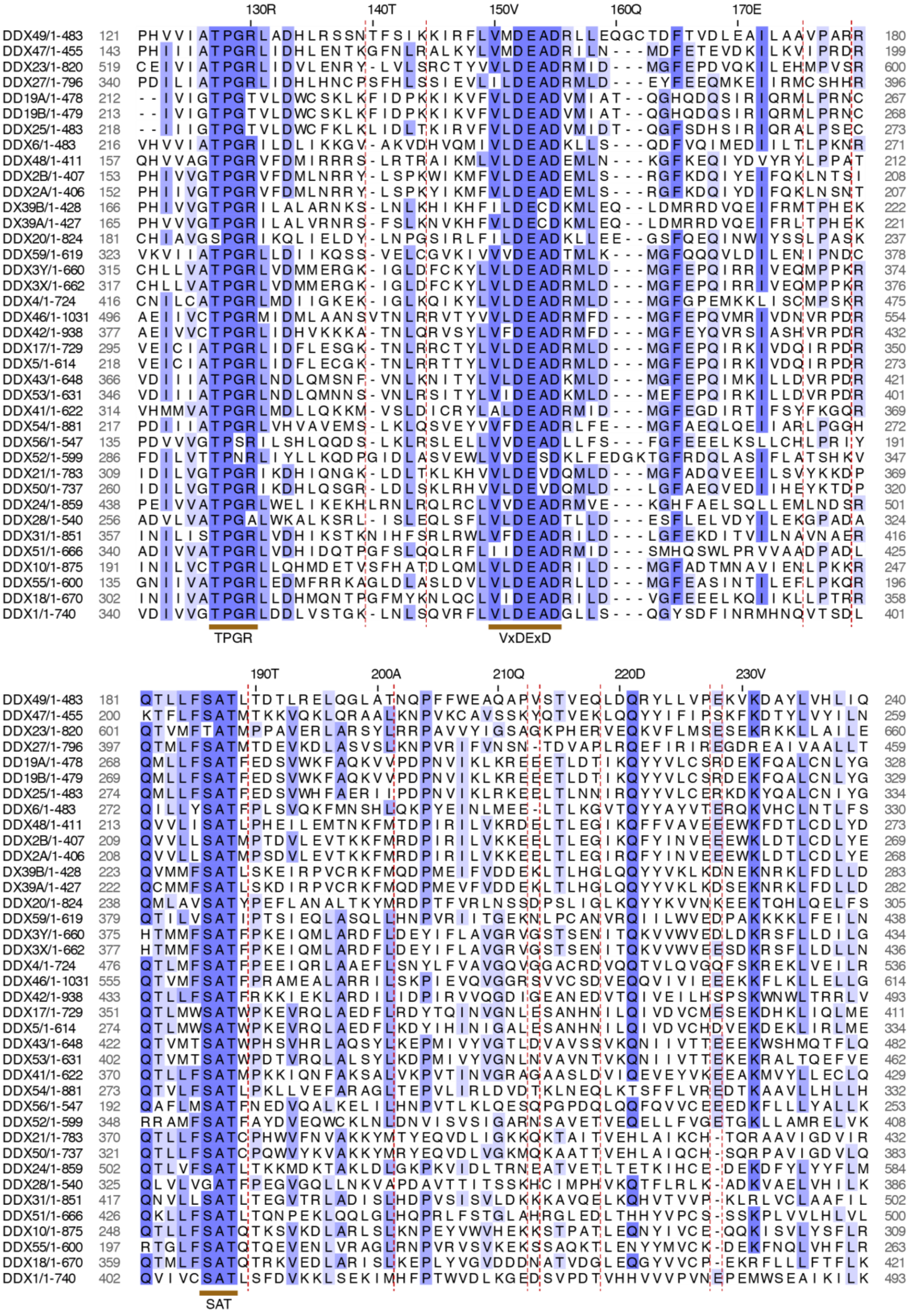
130R
140T
150V
160Q
170E
TPGR
VxDExD
190T
200A
210Q
220D
230V
SAT

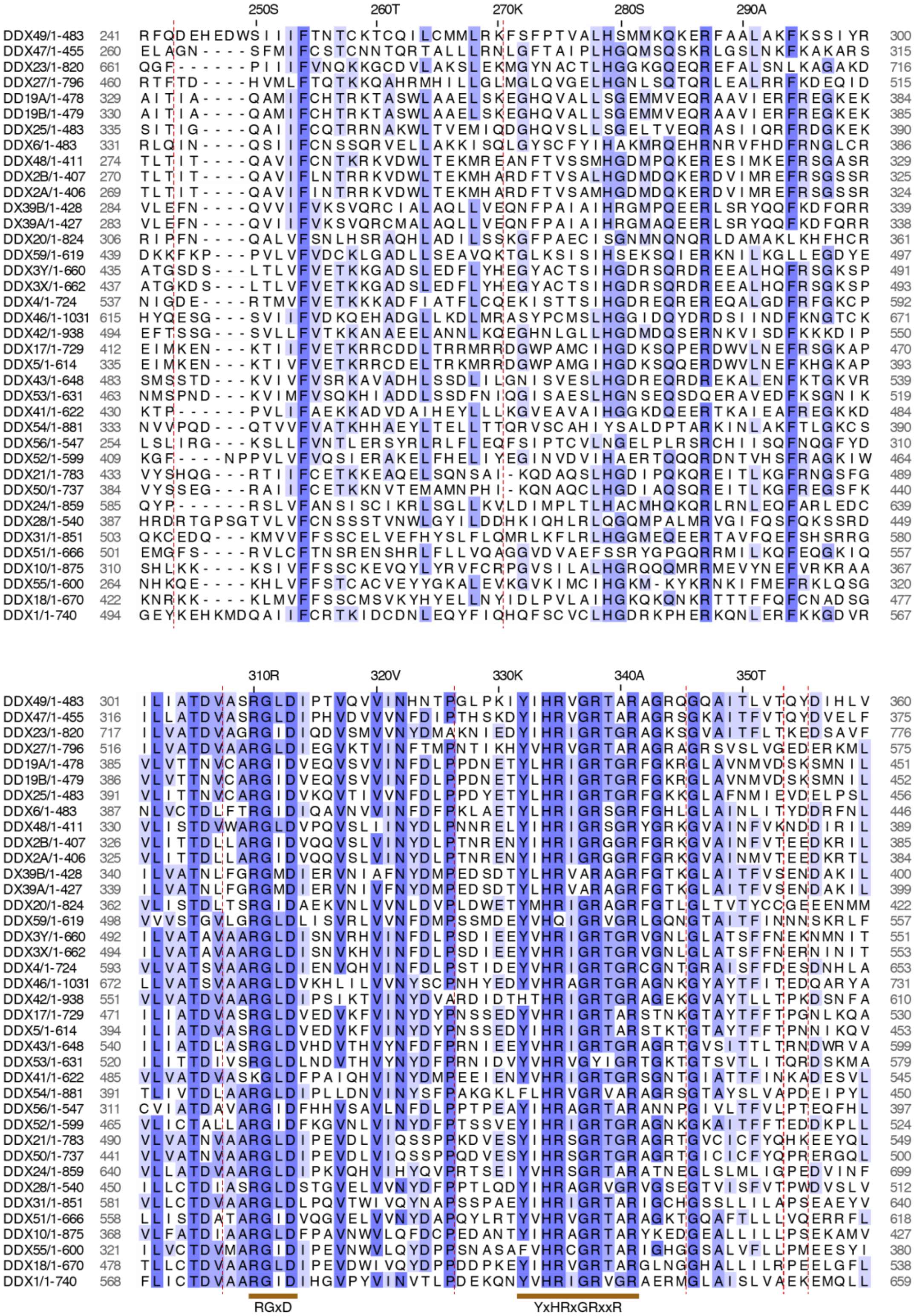
250S
260T
270K
280S
290A
310R
320V
330K
340A
350T
RGxD
YxHRxGRxxR

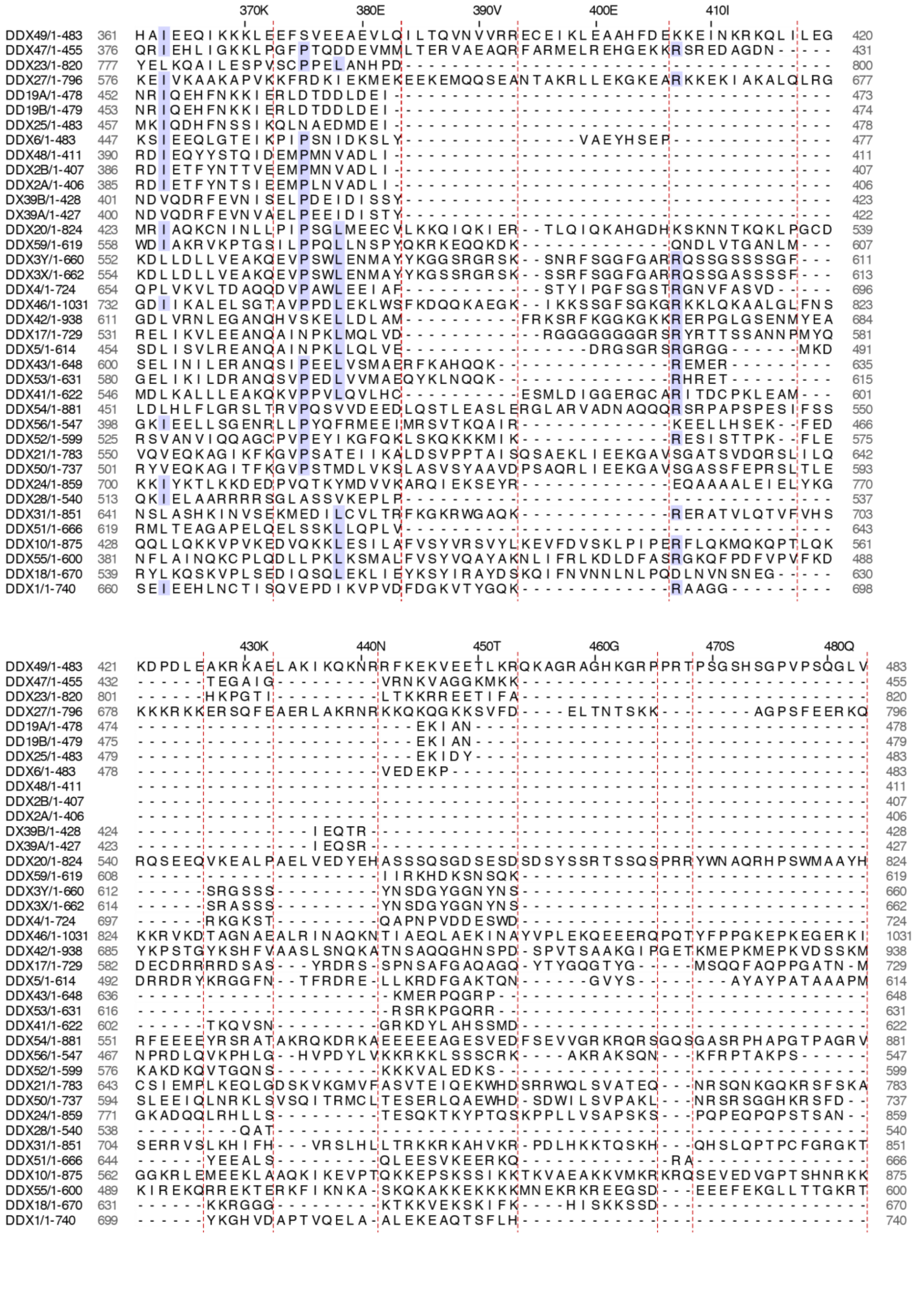

Supplementary Figure S1. Multiple sequence alignment of human DExD box helicases

Amino acid sequences of 38 human DExD box helicases (Supplementary Table S1) were obtained from Uniprot database. Multiple sequence alignment (MSA) was generated using MAFFT (1) from EMBL-EBI server. In the MSA provided, DDX49

was set as the reference sequence and all insertions are masked. The positions of insertions are shown by vertical dotted red lines. Conserved residue columns with sequence identity > 40% are highlighted in blue color, with darker color indicating higher conservation. The grey numbers indicate the start and end of the specific DExD box helicase for a given section of MSA. The number followed by amino acid at the top of the alignment corresponds to the residue position in the reference, DDX49. Sequence motifs, predicted based on conservation profiles, are provided at the bottom of the MSA with a brown line.

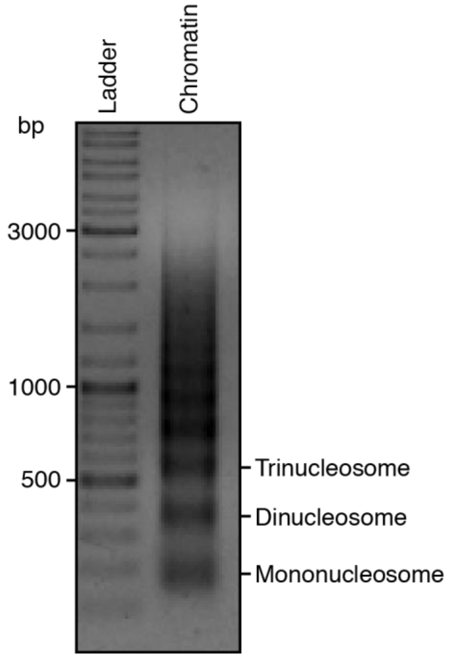

Supplementary Figure S2. Chromatin preparation from HEK293 cells

The sheared chromatin was run on 1% agarose gel and stained with ethidium bromide. The gel shows the presence of mononucleosomes, dinucleosomes, trinucleosomes and higher molecular weight chromatin.

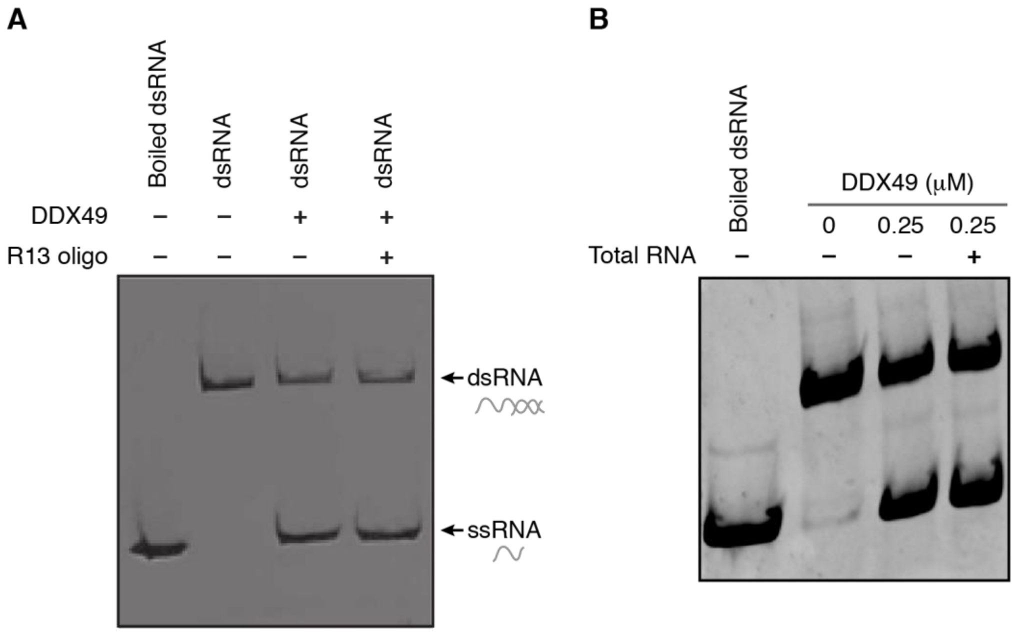

Supplementary Figure S3. The helicase activity of DDX49 is RNA independent

RNA helicase activity of DDX49 was determined in the presence and absence of R13 RNA oligo (**A**) or Total RNA (**B**).

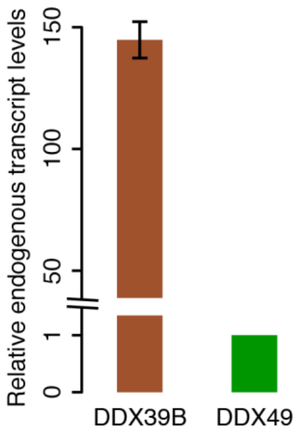

Supplementary Figure S4. Relative quantification of endogenous DDX39B and DDX49 transcript levels

Quantitative RT-PCR analysis of DDX39B and DDX49 levels in HEK293 cells. The levels of DDX39B and DDX49 were normalized to GAPDH expression and are presented relative to the DDX49 expression. Data are represented as mean of three independent experiments, with error bars representing standard deviation.

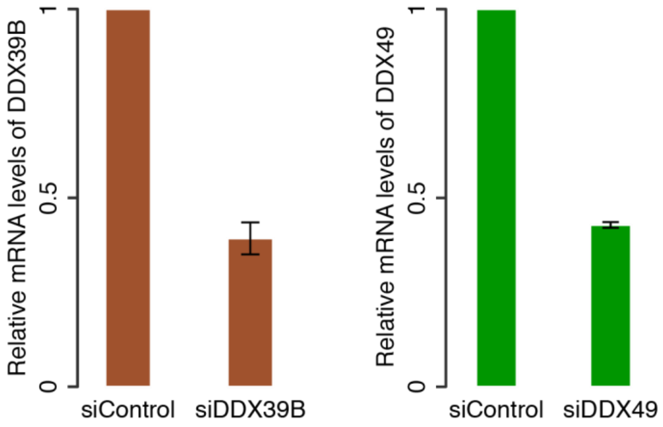

Supplementary Figure S5. Efficiency of siRNA mediated knockdown of DDX39B and DDX49

Efficiency of siRNA mediated knockdown of DDX39B or DDX49 transcripts was tested using quantitative RT-PCR analysis in HeLa cells. Cells were transfected with control siRNA, DDX39B siRNA (left panel) or DDX49 siRNA (right panel). The transcript levels of DDX39B and DDX49 were normalized to GAPDH expression and are presented relative to the control sample. Data are represented as mean of three independent experiments, with error bars representing standard deviation.

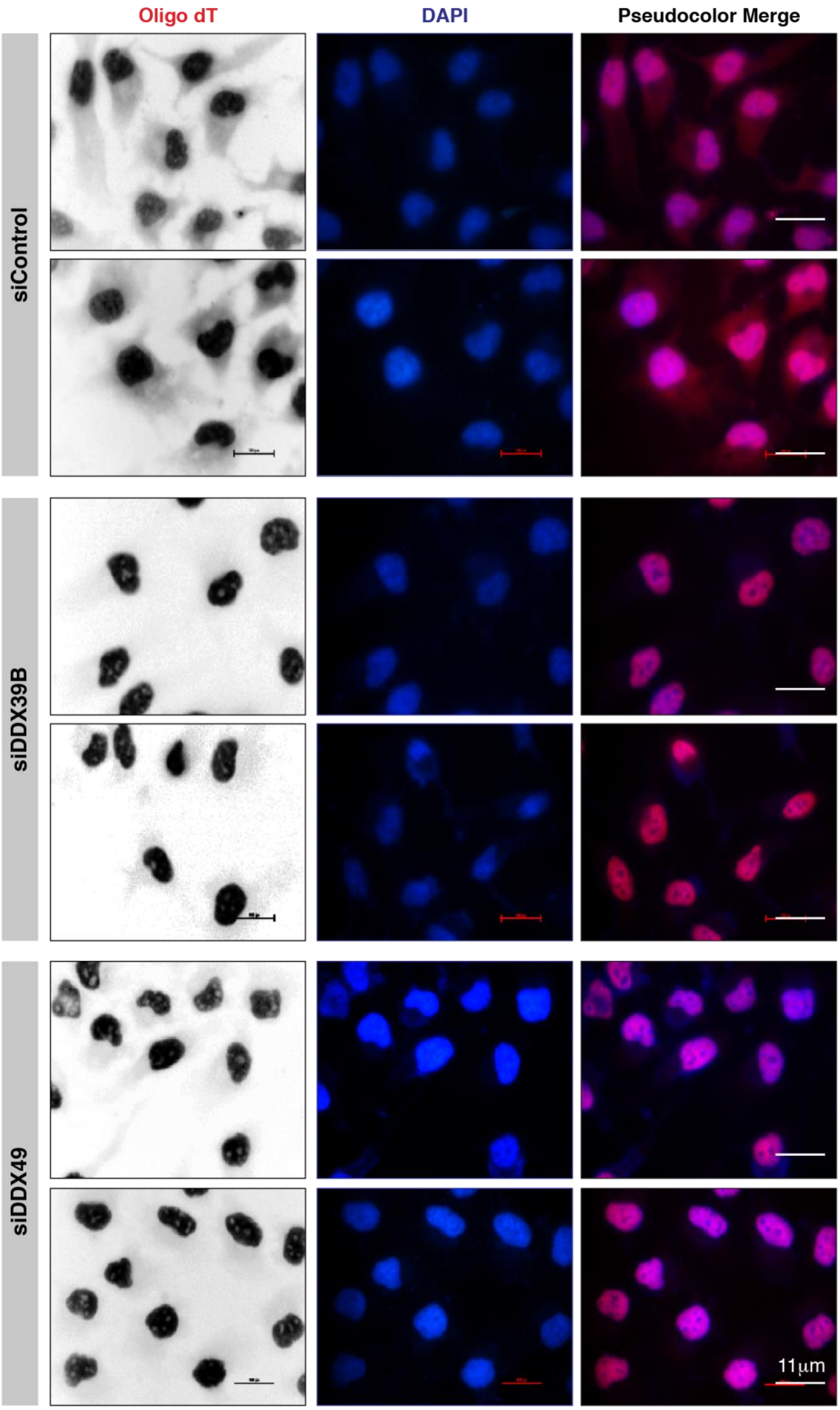

Oligo dT
DAPI
Pseudocolor Merge
siControl
siDDX39B
siDDX49
11µm

Supplementary Figure S6. DDX49 is essential for efficient poly (A)$^+$ RNA export

HeLa cells treated with control, DDX39B or DDX49 siRNA were fixed and poly (A)$^+$ RNAs were labelled with ATTO 550 dye (red in merge; inverted grey image in monochrome channel). Subsequently, cells were washed with SSC buffer and DNA stained with DAPI (blue) and embedded in Mowiol. Images were acquired using fluorescence microscope (Nikon-Eclipse 80i) with 100X oil immersion objective. Scale bar is depicted.

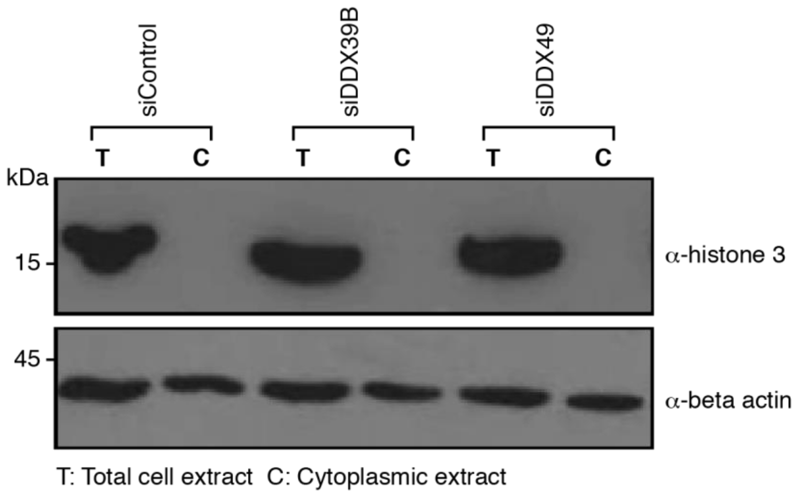

Supplementary Figure S7. The purity of the cytoplasmic extract used for the quantification of mRNA export (Corresponds to Figure 2C)

Western blot analysis of total (T) and cytoplasmic (C) extracts prepared from HeLa cells, which were transfected with control siRNA, DDX39B siRNA or DDX49 siRNA. The immunoblotting was performed with histone 3 antibody (nuclear protein) or beta actin antibody. The absence of histone 3 signal in cytoplasmic extracts indicates the absence of nuclear leakage in the cytoplasmic fractions.

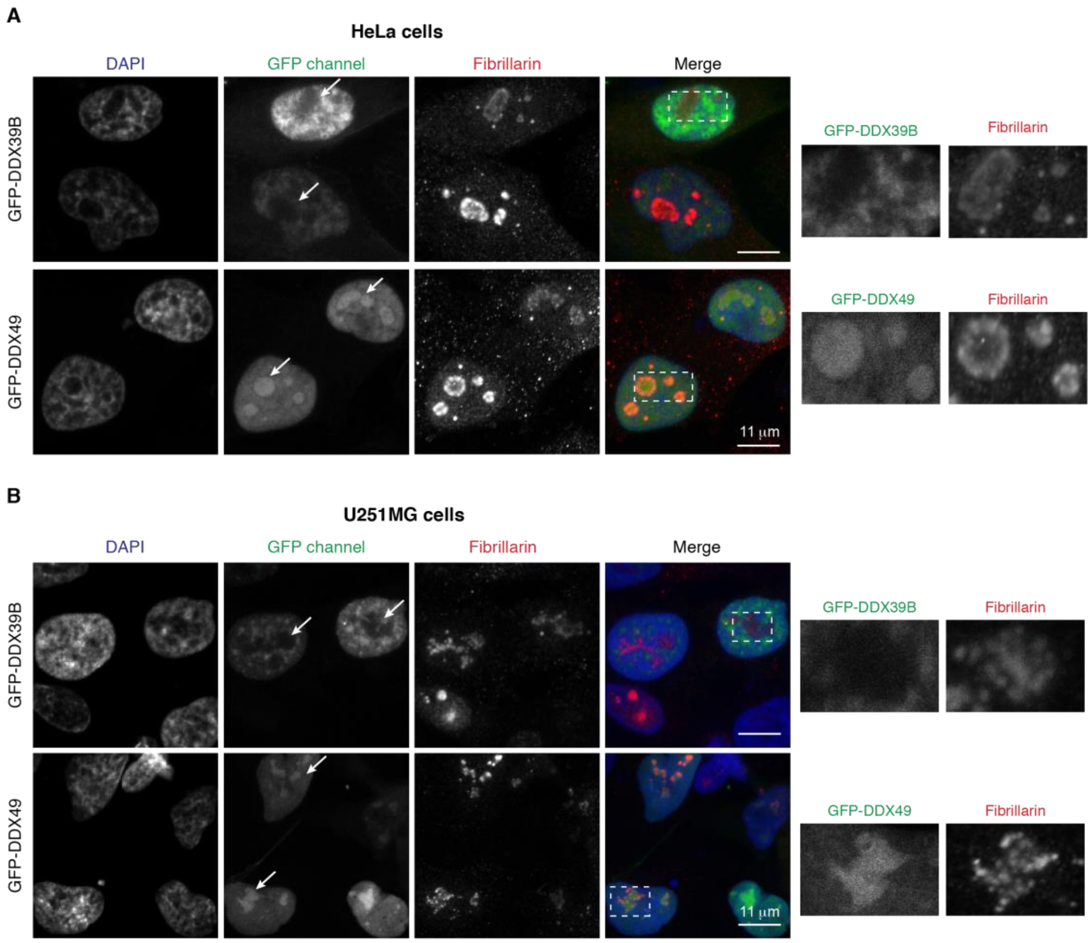

Supplementary Figure S8. Subcellular localization of DDX49 and DDX39B in different cell types

DDX49 is localized in the nucleolus. Representative images depicting the co-localization of DDX49 and fibrillarin in (**A**) HeLa cells and (**B**) U251MG cells. Cells were transfected with pEGFP-DDX49 or pEGFP-DDX39B (green), immunostained with fibrillarin antibody (red), which stains the nucleolus. DAPI is shown in blue. Representative image depicting the colocalization of DDX49 and fibrillarin and the absence of colocalization of DDX39B with fibrillarin. The white arrows indicate the nucleolus. Insets show the magnified images of the regions outlined by the dotted white squares. Scale bar is depicted.

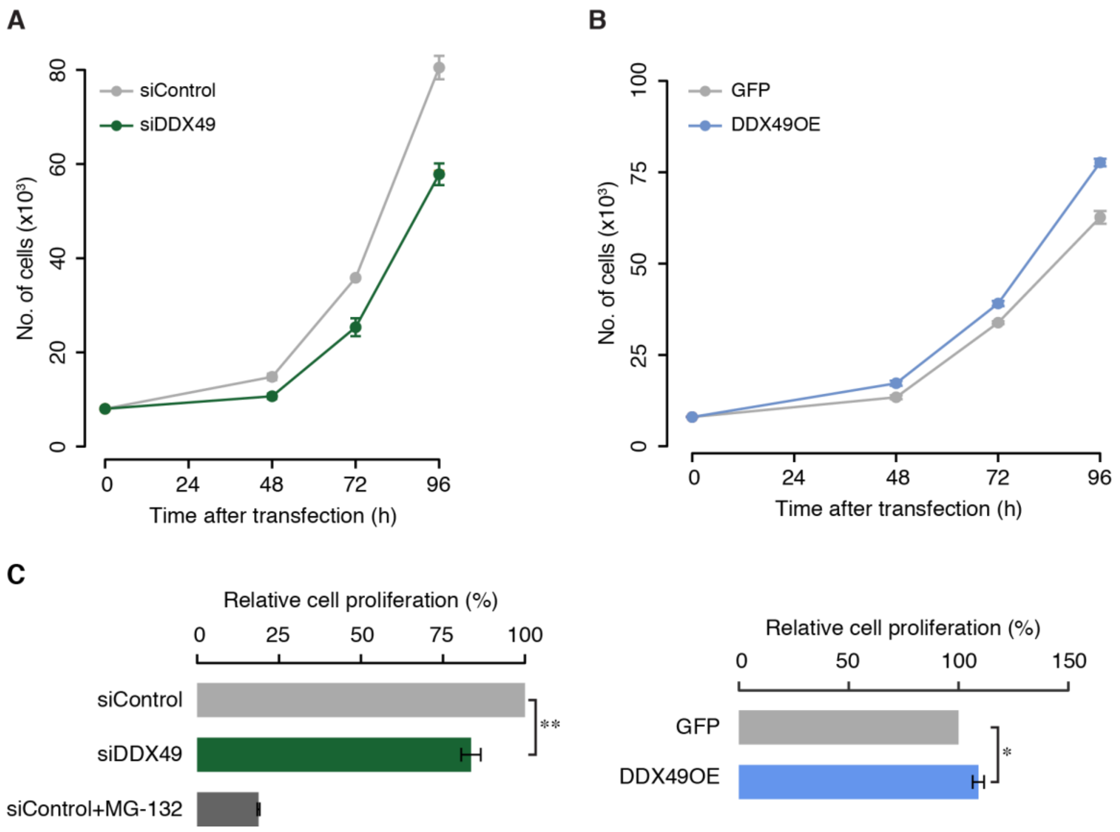

Supplementary Figure S9. Effect of DDX49 perturbation on cell proliferation

(**A**) Depletion of DDX49 (siDDX49) inhibits cell proliferation. HeLa cells were transfected with control siRNA or DDX49 siRNA and the cell numbers were counted at 48, 72 and 96 hours post transfection. The dots represent the mean of triplicates, with error bars representing standard deviation. (**B**) Overexpression of DDX49 (DDX49OE) promotes cell proliferation. HeLa cells were transfected with pEGFP-C1 vector or pEGFP-DDX49 and the cell numbers were counted 48, 72 and 96 hours post transfection. The dots represent the mean of triplicates, with error bars representing standard deviation. (**C**) HeLa cells were transfected with the control or DDX49 siRNAs or treated with 0.5 µg of MG-132 (left panel) or pEGFP-DDX49 (right panel). After 72 hours of transfection or drug treatment, MTT assay was performed. MG-132 is a potent proteosomal inhibitor known to induce apoptosis (2,3) was used as positive control for the assay. Data are represented as mean of three independent experiments, with error bars representing standard deviation. Statistical significance was estimated using two-tailed T-test. * indicates $P<0.05$ and ** indicates $P<0.01$.

A

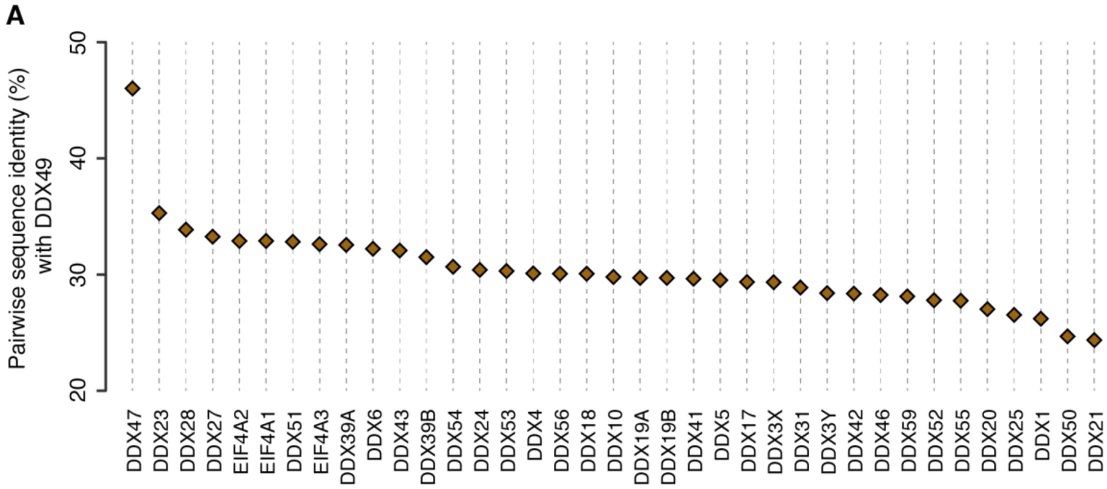

B

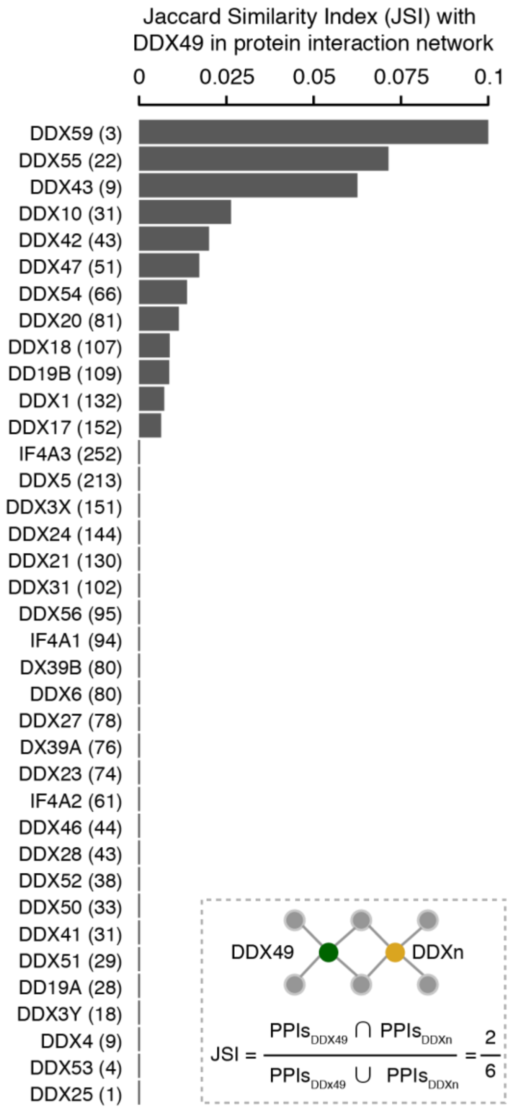

$$JSI = \frac{PPIs_{DDX49} \cap PPIs_{DDXn}}{PPIs_{DDX49} \cup PPIs_{DDXn}} = \frac{2}{6}$$

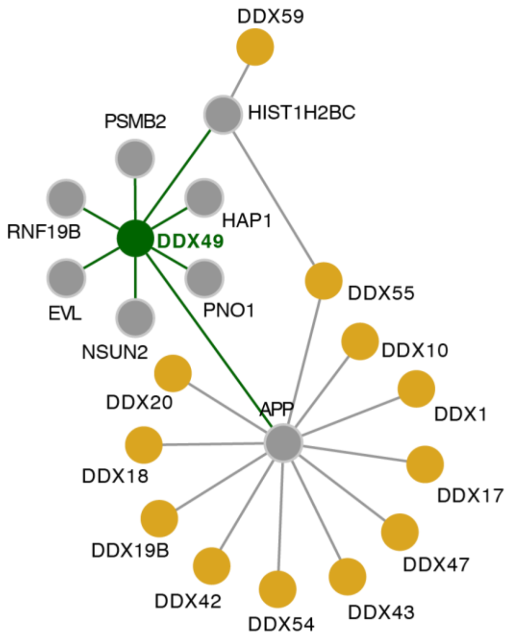

DDX49 interactor	Description
APP	Amyloid-beta A4 protein
NSUN2	tRNA (cytosine(34)-C(5))-methyltransferase
EVL	Ena/VASP-like protein
RNF19B	E3 ubiquitin-protein ligase RNF19B
PSMB2	Proteasome subunit beta type-2
HAP1	Huntingtin-associated protein 1
PNO1	RNA-binding protein PNO1
HIST1H2BC	Histone H2B type 1-C/E/F/G/I

Supplementary Figure S10. DDX49 is a distinct DExD box RNA helicase

(**A**) Pairwise sequence identity of DDX49 with the other DExD box RNA helicases. (**B**) Protein-protein interaction landscape of DDX49. The bar plot on the left shows the extent of overlap of interaction partners between DDX49 and other DExD box RNA helicases, quantified using Jaccard Similarity Index (JSI). The legend at the bottom provides a schematic representation of how JSI was calculated. The numbers in the parenthesis represent the number of protein interactors identified so far for the corresponding DExD box helicase. The network on the right, drawn using Cytoscape, shows the interaction of DDX49 interactors with diverse DExD box RNA helicases. DDX49 (green color node) and its interaction partners (grey nodes) are connected by green color links. Golden yellow nodes represent the other DExD box RNA helicases and the grey links show their connections with the interaction partners of DDX49. The table below the network provides a brief description of the proteins interacting with DDX49.

Supplementary Table S1. Members of human DExD family of RNA helicases

Uniprot accession	Ensembl gene	Entrez gene	Gene name(s)	Protein length	DExD Motif type	DExD Motif position
Q9H8H2	ENSG00000125485	64794	DDX31	851	DEAD	388-391
O15523	ENSG00000067048	8653	DDX3Y, DBY	660	DEAD	345-348
Q9NUU7	ENSG00000168872	55308	DDX19A, DDX19L	478	DEAD	241-244
Q9UMR2	ENSG00000157349	11269	DDX19B, DBP5, DDX19, TDBP	479	DEAD	242-245
Q92499	ENSG00000079785	1653	DDX1	740	DEAD	370-373
Q7L014	ENSG00000145833	9879	DDX46, KIAA0801	1031	DEAD	529-532
Q9Y6V7	ENSG00000105671	54555	DDX49	483	DEAD	152-155
Q13838	ENSG00000198563	7919	DDX39B, BAT1, UAP56	428	DECD	196-199
Q13206	ENSG00000178105	1662	DDX10	875	DEAD	222-225
Q9NVP1	ENSG00000088205	8886	DDX18, cPERP-D	670	DEAD	333-336
Q9GZR7	ENSG00000089737	57062	DDX24	859	DEAD	471-474
Q96GQ7	ENSG00000124228	55661	DDX27, cPERP-F, RHLP, HSPC259, PP3241	796	DEAD	371-374
O00571	ENSG00000215301	1654	DDX3X, DBX, DDX3	662	DEAD	347-350
Q9NXZ2	ENSG00000080007	55510	DDX43, HAGE	648	DEAD	396-399
Q86TM3	ENSG00000184735	168400	DDX53, CAGE	631	DEAD	376-379
Q8NHQ9	ENSG00000111364	57696	DDX55, KIAA1595	600	DEAD	171-174
Q5T1V6	ENSG00000118197	83479	DDX59, ZNHIT5	619	DEAD	353-356
Q9NR30	ENSG00000165732	9188	DDX21	783	DEVD	339-342
Q9UHL0	ENSG00000109832	29118	DDX25, GRTH	483	DEAD	247-250
Q9UJV9	ENSG00000183258	51428	DDX41, ABS	622	DEAD	344-347
Q9Y2R4	ENSG00000278053	11056	DDX52, ROK1, HUSSY-19	599	DESD	318-321
Q9UHI6	ENSG00000064703	11218	DDX20, DP103, GEMIN3	824	DEAD	211-214

Uniprot accession	Ensembl gene	Entrez gene	Gene name(s)	Protein length	DExD Motif type	DExD Motif position
Q9NUL7	ENSG00000182810	55794	DDX28, MDDX28	540	DEAD	286-289
Q9H0S4	ENSG00000213782	51202	DDX47	455	DEAD	174-177
Q9NQI0	ENSG00000152670	54514	DDX4, VASA	724	DEAD	446-449
Q9BQ39	ENSG00000107625	79009	DDX50	737	DEVD	290-293
Q8TDD1	ENSG00000123064	79039	DDX54	881	DEAD	247-250
P26196	ENSG00000110367	1656	DDX6, HLR2, RCK	483	DEAD	246-249
Q86XP3	ENSG00000198231	11325	DDX42	938	DEAD	407-410
Q8N8A6	ENSG00000185163	317781	DDX51	666	DEAD	371-374
Q92841	ENSG00000100201	10521	DDX17	729	DEAD	325-328
Q9BUQ8	ENSG00000174243	9416	DDX23	820	DEAD	549-552
Q9NY93	ENSG00000136271	54606	DDX56, DDX21, NOH61	547	DEAD	166-169
P17844	ENSG00000108654	1655	DDX5, G17P1, HELR, HLR1	614	DEAD	248-251
O00148	ENSG00000123136	10212	DDX39A, DDX39	427	DECD	195-198
P38919	ENSG00000141543	9775	EIF4A3, DDX48, KIAA0111	411	DEAD	187-190
Q14240	ENSG00000156976	1974	EIF4A2, DDX2B, EIF4F	407	DEAD	183-186
P60842	ENSG00000161960	1973	EIF4A1, DDX2A, EIF4A	406	DEAD	182-185

Supplementary Table S2. List of the siRNAs used in various experiments

**siRNA**	**Sequence (5' to 3') (Sense strand)**
siControl	AUCCGCGCGAUAGUACGUA
siDDX49	GAGAGUGUGAGAUCAAACU
siDDX39B	GUGCUACCUUGAGCAAAGA

Supplementary Table S3. List of the primers used in qPCR for various investigations

**Gene Name**	**Sequence (5' to 3')**	**Investigation**
U1 snRNA-FP	ACCTGGCAGGGGAGATACCA	mRNA Export
U1 snRNA-RP	GGGGAAAGCGCGAACGCAGT	mRNA Export
Egr1-FP	GACCCGTTCGGATCCTTTCC	mRNA Export
Egr1-RP	GCCACAAGGTGTTGCCACTG	mRNA Export
GAPDH-FP	TCACCAGGGCTGCTTTTAAC	mRNA Export
GAPDH-RP	TGACGGTGCCATGGAATTTG	mRNA Export
YY2-FP	TATAGCGGCTGCGAAAAGAT	mRNA Export
YY2-RP	CTTTGCCACATTCTGCACAT	mRNA Export
47S rRNA-FP	CTGTCCTCTGGCGACCTG	47S rRNA level
47S rRNA-RP	GAGAGAACAGCAGGCCCG	47S rRNA level
DDX39B-FP	GAGCAAAGAGATCCGTCCAG	DDX39B Expression
DDX39B-RP	TTCCGGTTCTTCTCGTTGTC	DDX39B Expression
DDX49-FP	CTTCTTCTGGGAAGCACAGG	DDX49 Expression
DDX49-RP	TTCATCATGGAGTGCAGAGC	DDX49 Expression
H42-FP	GCACCGTTTGTGTGGGGTTGG	ChIP
H42-RP	CGAGACAGATCCGGCTGGCAG	ChIP
H0-FP	GGAGGTATATCTTTCGCTCCGAG	ChIP
H0-RP	GACGACAGGTCGCCAGAGGA	ChIP
H13-FP	ACCTGGCGCTAAACCATTCGT	ChIP
H13-RP	GGACAAACCCTTGTGTCGAGG	ChIP
H18-FP	GTTGACGTACAGGGTGGACTG	ChIP
H18-RP	GGAAGTTGTCTTCACGCCTGA	ChIP

Supplementary References

1. Katoh, K. and Standley, D.M. (2013) MAFFT multiple sequence alignment software version 7: improvements in performance and usability. *Molecular biology and evolution*, 30, 772-780.

2. Shinohara, K., Tomioka, M., Nakano, H., Tone, S., Ito, H. and Kawashima, S. (1996) Apoptosis induction resulting from proteasome inhibition. *The Biochemical journal*, 317 (Pt 2), 385-388.

3. Grimm, L.M., Goldberg, A.L., Poirier, G.G., Schwartz, L.M. and Osborne, B.A. (1996) Proteasomes play an essential role in thymocyte apoptosis. *The EMBO journal*, 15, 3835-3844.
